# Quantitative analysis of sensitivity to a Wnt3a gradient in determination of the pole‐to‐pole axis of mitotic cells by using a microfluidic device

**DOI:** 10.1002/2211-5463.12525

**Published:** 2018-11-09

**Authors:** Takumi Hiraiwa, Yuichiro Nakai, Takahiro G. Yamada, Ryuichi Tanimoto, Hiroshi Kimura, Yoshinori Matsumoto, Norihisa Miki, Noriko Hiroi, Akira Funahashi

**Affiliations:** ^1^ Department of Biosciences and Informatics Keio University Yokohama Japan; ^2^ Department of Mechanical Engineering Tokai University Hiratsuka Japan; ^3^ Department of Applied Physics and Physico‐Informatics Keio University Yokohama Japan; ^4^ Department of Mechanical Engineering Keio University Yokohama Japan; ^5^ Department of Pharmacy Sanyo‐Onoda City University Japan

**Keywords:** microfluidics, neuroblastoma, pole‐to‐pole axis, quantitative gradient ODF2, Wnt3a

## Abstract

Proper determination of the cell division axis is essential during development. Wnt3a is a known regulator of the cell division axis; however, the sensitivity of cells to Wnt3a signalling and its role in determining the cell division axis have not been measured to date. To address this gap, we took advantage of the asymmetric distribution of outer dense fibre 2 (ODF2/cenexin) proteins on centrosomes in dividing cells. To precisely quantify the sensitivity of cells to Wnt3a signalling, we developed a microfluidic cell culture device, which can produce a quantitative gradient of signalling molecules. We confirmed that mitotic SH‐SY5Y neuroblastoma cells could detect a 2.5 ~ 5 × 10^−3^ nm·μm^−1^ Wnt3a concentration gradient and demonstrated that this gradient is sufficient to affect the determination of the pole‐to‐pole axis of cell division during the later stages of mitosis.

**Abbreviations**
Dkk1: dickkopf‐1
ES cell: embryonic stem cell
LRP5/6: lipoprotein receptor‐related protein 5 or 6
MPC: poly(2‐methacryloyloxyethyl phosphorylcholine‐co‐n‐butyl methacrylate)
ODF2: outer dense fibre 2
Wnt3a‐CM: Wnt3a‐containing medium

Determination of the cell division axis is an essential process in the development of various species 1, 2, 3, 4, 5 and is thought to be controlled by the extracellular environment, such as the niche formed by neighbouring stem cells, which contributes to cell maintenance and regulation 3, 5, 6. Previous work has confirmed that Wnt3a‐coated beads induce an asymmetric distribution of pluripotency proteins and direct the asymmetric inheritance of centrosomes in embryonic stem (ES) cells at the single‐cell level 7. However, there has been no research to date ascertaining the extent of the Wnt concentration gradient required to determine the cell division axis. Consequently, the quantitative threshold of cell sensitivity to Wnt signalling has not been ascertained.

We and others have shown the significance of quantitative analyses in understanding the pathways that precisely determine the cell division axis 8, 9. The long‐term goal of such analyses is to develop a precise control method to determine the cell division axis and, in turn, control daughter cell position, fate and differentiation, a method that will be useful in regenerative medicine.

Wnt proteins are secreted proteins, which are evolutionarily well conserved among multicellular eukaryotes, from insects to animals. Wnt proteins play crucial roles in the organisation of tissues 10, 11, 12, 13, control of the cell cycle 10, 14, 15 and differentiation of stem cells 14, 16. When a Wnt ligand binds to a Frizzled receptor in conjunction with low‐density lipoprotein receptor‐related protein 5 or 6 (LRP5/6) co‐receptor to form a ternary complex, Wnt signalling is activated in cells 17. Wnt proteins form a region‐specific concentration gradient by tethering to the plasma membrane of secreted cells and the extracellular matrix of tissues 11, 12, 13, 18. This region‐specific concentration gradient surrounding mitotic cells may change their fate after cell division, by determining the cell division axis. For example, localised Wnt signalling determines the axis of asymmetric cell division of embryonic endomesodermal cells in *C. elegans* and ES cells 3, 7. In these cases, the concentration gradient of Wnt and the difference in the number of Wnt‐Frizzled pairs on the opposite sides of a cell may significantly influence its cellular fate. In this study, we used Wnt3a protein to activate Wnt signalling since Wnt3a can determine the axis of asymmetric cell division in ES cells, whereas Wnt5a does not 7.

Neuroblastoma cells have a potential for differentiation into neural cells through asymmetric cell division 19, 20. This behaviour supposedly mimics the cells in a neural crest. During asymmetric cell division of human neuroblastoma cells, the daughter centrosome with the granddaughter centriole is inherited in one daughter cell, which expresses NuMA, whereas the mother centrosome with the grandmother centriole is inherited in the other daughter cell 20. This example indicates that neuroblastoma cells are equipped with the mechanisms required to determine the cell division axis. However, these mechanisms have not been fully elucidated. In this study, we chose the SH‐SY5Y neuroblastoma cell line 20.

To elucidate the effect of spatially biased Wnt signalling on the division of SH‐SY5Y cells, we developed a microfluidic device, which establishes a spatiotemporally stable concentration gradient of solutes in the cell culturing space. A microfluidic device is a tool equipped with microchannels. The fluid dynamic properties of liquids in a microchannel are different from those of a bulk cell culture system. For example, solute concentration gradients are predictably formed by molecular diffusion. This property of a fluid in a microchannel allows the formation of a quantitative concentration gradient of solutes to stimulate cells.

To distinguish the polarity axis of the dividing cell, we observed the asymmetric distribution of ODF2, also known as the splicing variant cenexin (ODF2/cenexin). ODF2/cenexin is a pericentriolar protein and is essential for the formation of distal and subdistal appendages on the centriole. A grandmother centriole inherits ODF2/cenexin primarily during mitosis 21, 22, and this asymmetric inheritance of mother centrioles during mitosis determines the asymmetric cell division axis 7, 23, 24.

We investigated how the concentration gradient of Wnt3a during mitosis determines the orientation of the pole‐to‐pole axis (Fig. 1). We found that the pole‐to‐pole axis in mitotic SH‐SY5Y cells is determined by the concentration gradient of Wnt3a before metaphase–anaphase transition, with a minimum Wnt3a concentration threshold of 2.5 × 10^−3^nm·μm^−1^. This indicates that a low concentration gradient of signalling molecules in the culturing environment of mitotic SH‐SY5Y cells is sufficient to determine the axes of the asymmetric distribution of mitotic factors that control metaphase spindle orientation. Thus, this study provides a quantitative framework to study the extracellular factors that can control the intracellular events important for regenerative medicine applications.

**Figure 1:**
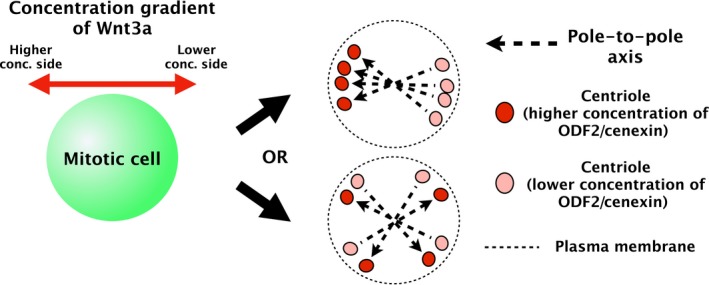
Pole‐to‐pole axis in a mitotic cell. The pole‐to‐pole axis in this study is indicated by a black dashed line with an arrow, which connects two centrioles and is orientated towards higher ODF2/cenexin concentrations. If the axis is determined by the Wnt3a concentration gradient, the axis may be aligned as indicated in the upper right figure. However, if the axis is not affected by the Wnt3a concentration gradient, the axis will be randomly orientated, as shown in the lower right figure.

## Materials and methods

### Mask design

Our device consists of three layers: the cell culturing layer, the fluidic layer and pneumatic layer. The cell culturing layer consists of microgrooves (width: 250 μm, height: 30 μm) and a main channel (width: 1000 μm, height: 160 μm), and the fluidic layer consists of lower and higher channels. The pneumatic layer consists of an air valve 25. The five film masks (microgrooves, main channel, lower channel, higher channel and air valve) were designed using Inkscape (version 0.48, http://www.inkscape.org) and purchased from Vanfu Inc. (Tokyo, Japan).

### Device fabrication

SU‐8 3010 (Newton, MA, USA) was applied to a glass wafer (S9111, Matsunami Glass, Osaka, Japan), which was then spun and baked at 100 °C. The wafer was exposed to UV through the microgrooves mask using a desktop aligner (EMA‐400, Union Optical, Tokyo, Japan), then baked at 65 and 100 °C. After baking, SU‐8 3050 was applied to the wafer, and it was spun again and baked at 100 °C. The main channel mask was aligned on the wafer, then exposed to UV and baked at 65 and 100 °C. The wafer was then soaked in SU‐8 developer. The parameters of the processes described above are listed in Table S1.

A PDMS mixture of a prepolymer and curing agent at a ratio of 10 : 1 was poured into a plastic case containing the master mould. The depth of the cell culturing layer, the fluidic layer and the pneumatic layer was approximately 2, 0.5 and 5 mm, respectively. A thin PDMS layer (0.5 mm) was fabricated according to a previously published research method 26. The layers were sonicated in ethanol for 5 min, then dried under a stream of nitrogen and on a hot plate at 100 °C. The surface of the fluidic layer and the pneumatic layer was activated by a soft plasma etcher (SEDE‐P, Meiwa Fosis, Tokyo, Japan). After plasma treatment, the treated surface of the fluidic layer was immediately placed in contact with the plasma‐treated surface of the pneumatic layer. The two contacting layers were then baked at 100 °C for several minutes. After bonding the fluidic layer to the pneumatic layer, fluidic inlets were punched using a biopsy punch, and then, the process above was repeated for the cell culturing layer. After bonding all the layers, the cell inlet and outlet were punched using a biopsy punch. The inner surfaces of the microchannel were coated with 0.5 wt% poly(2‐methacryloyloxyethyl phosphorylcholine‐co‐n‐butyl methacrylate) polymer (MPC polymer; Lipidure‐CM5206, NOF) in absolute ethanol (EtOH)/chloroform (v/v = 1/9) 27.

### Evaluation of blocking nonspecific protein adsorption on PDMS surface

We coated a PDMS microchannel (width: 1 mm, height: 200 μm) with 0.5 wt% MPC polymer in EtOH/chloroform 27. A culture medium containing fluorescein isothiocyanate‐BSA (FITC‐BSA; 0.23 mg·mL^−1^, Sigma, Tokyo, Japan) was perfused in the straight microchannel at a flow rate of 5 μL·min^−1^ for 3 h. After perfusion, we rinsed the microchannel with ultrapure water and measured the intensity of FITC on the inner surface using confocal microscopy. We also evaluated the endurance of the blocking by repeating the fixation, ethanol wash and heat sterilisation procedures several times.

### Evaluation of the concentration gradient in the device

To predict the concentration of Wnt3a at a specific cell position in a stimulation experiment, we derived a prediction based on experimental data. We set up an experimental system that was identical to the experimental cell culturing set‐up. We observed the concentration gradient in the cell culturing channel formed by 0.1 mg·mL^−1^ FITC conjugated with dextran (40 kDa, FD40S, Sigma) in Opti‐MEM (Life Technologies, Tokyo, Japan) at a flow rate of 1.0, 2.0 and 3.0 μL·min^−1^. Before acquiring an image of the concentration gradient of FITC‐dextran, we acquired an image of the cell culturing channel filled with FITC‐dextran solution to correct the intensity value. To correct the intensity value, the intensity value in the concentration gradient image was divided by normalised values from the image. After correcting the image, we measured the intensity in the regions of interest (ROIs; width 15 μm and length 900 μm approximately). The width and the length of the ROIs is approximately the radius of the mitotic cells and the width of the cell culturing channel, respectively. The ROIs were set vertically from wall of the cell culturing channel. The concentration gradient across the middle of the culturing channel was fitted according to the following equation 28, 29:

(1) $$C\left(x\right)=\frac{1}{2}\left(C_{\max}-C_{\min}\right)\text{erfc}\left(\frac{x-\alpha }{\sqrt{\beta }}\right)+C_{\min}$$

where *C* is the normalised FITC‐dextran concentration ranging from background (= 0) to maximum intensity (= 1) as a function of position *x*;* C*_max_, *C*_min_, α and β are coefficients. The *C*_max_ and *C*_min_ coefficients are the maximum and minimum intensity in the ROI, respectively. The coefficient α is the estimated position where is $C=1/2\left(C_{\max}-C_{\min}\right)+C_{\min},\;\beta$ is the mean square displacement of molecules, and erfc is the complementary error function. We calculated erfc using the NORMT3 package installed in R 30. The α and β coefficients were estimated with the Nonlinear Least Square (nls) function using R. Furthermore, the fluctuation of the normalised concentration difference (σ) was evaluated with the following equation:

(2) $$\sigma =\frac{\sum_{i=0}^{N}\left|\Delta C_{1i}-\text{Median}\left(\Delta C_{1}\right)\right|}{N}$$

where *N* is the total number of images, Δ*C*_1_ is the normalised concentration difference (see Section, Development and evaluation of the microfluidic device) at each position (*x *=* *200, 400 and 600 μm) in the channel (channel no. = 1), and Δ*C*_1i_ is the normalised concentration difference of the ith image. Images were sequentially acquired at intervals of 1, 10, 100, and 1000 s.

### Cell culture

We obtained an SH‐SY5Y cell line, which is a thrice‐cloned subline of the bone marrow biopsy‐derived line SK‐N‐SH, from the European Collection of Cell Cultures Cell Bank (Cat. No. is 94030304). Cell culture reagents for SH‐SY5Y cells were obtained from Wako Pure Chemical Industries (Osaka, Japan). SH‐SY5Y cells were routinely cultured in Dulbecco's modified Eagle medium/Ham's F‐12 Nutrient Mixture (1 : 1, Life Technologies) supplemented with 15% fetal bovine serum in a 5% CO_2_ incubator at 37 °C. For cell cycle synchronisation, double thymidine (2.0 mm) blocking was performed 31, 32. For immunofluorescence staining, cells were cultured on a glass bottom dish (IWAKI) coated with collagen (Cellmatrix Type IV, Nitta Gelatin).

Recombinant Wnt3a protein (5036‐WN, R&D Systems, Minneapolis, MN, USA) was dissolved at 200 μg·mL^−1^ in sterilised water containing 0.1% BSA (017‐22231, Wako, diluted 1 in 300 with sterilised ultrapure water) to obtain a stock solution. Recombinant Dickkopf‐1 (Dkk1) protein (5439‐DK, R&D Systems) was dissolved at 100 μg·mL^−1^ in phosphate buffered saline (PBS, 16‐23555 Wako). The working solution was adjusted by diluting the stock solution in the culture medium.

### Immunofluorescent staining

Rabbit polyclonal anti‐Wnt3a (ab28472, Abcam, Tokyo, Japan) and rabbit polyclonal anti‐LRP6 phosphorylated T1479 (Tp1479; PAB12632, Abnova, Taipei, Taiwan) were diluted in PBS at 1/200, and mouse monoclonal anti‐ODF2/cenexin (H00004957, Novus, Tokyo, Japan) was diluted in PBS at 1/500. Secondary antibodies, which were anti‐rabbit‐IgG (H+L) conjugated with Alexa Fluor 488 (Life Technologies) and anti‐mouse‐IgG (H+L) conjugated with Alexa Fluor 488 (Life Technologies), were diluted in PBS at 1/1000 32, 33.

For Tp1479 LRP6 staining, we followed previously published protocols 7. For ODF2/cenexin staining, cells were fixed for 5 min in −20 °C methanol before blocking with 3% BSA (012‐23881, Wako, BSA) in PBS for 30 min. The cells were incubated with mouse monoclonal anti‐ODF2/cenexin for 1 h at room temperature. The cells were then washed three times with PBS and incubated with an anti‐mouse‐IgG (H+L) secondary antibody conjugated with Alexa Fluor 488 for 1 h at room temperature in the dark. Nuclei were visualised with Hoechst 33342 (Lonza, Tokyo, Japan) at a concentration of 1 μg·mL^−1^ in PBS.

For Wnt3a staining, the Wnt3a protein was added to a glass bottom dish and incubated for 12 h. After 12 h, the dish was washed three times with PBS, before incubation with rabbit polyclonal anti‐Wnt3a for 1 h at room temperature. The dish was washed three times with PBS and incubated with an anti‐rabbit IgG (H+L) antibody conjugated with Alexa Fluor 488 for 1 h at room temperature in the dark.

To fix cells in the device, plugs and tubing were removed from each port after cell culture, and then, PBS was injected into the fluid inlets to wash out the medium. For ODF2/cenexin staining, −20 °C methanol was injected into the cell inlet, and then, the device was immersed in a 500‐mL beaker filled with −20 °C methanol for 5 min. After fixation, to wash out the methanol and block the cells, 3% BSA in PBS was injected into the fluid inlets and the fluid outlet. After blocking, the primary antibody was injected into the fluid inlets and the fluid outlet, then incubated at room temperature. After 1 h, PBS was injected into the fluid inlets and the fluid outlet, and then, the secondary antibody was injected before incubation at room temperature. After 1 h, the secondary antibody was washed out, and then, 1 μg·mL^−1^ Hoechst in PBS was injected. After 5 min, the Hoechst was washed out, and the cells were observed with a confocal microscope.

### Microscopic imaging and image analysis

Before quantification of the phosphorylation level of Tp1479 and ODF2/cenexin, the intensity was calibrated according to the objective height, since the different refractive index between the objective lens and ODF2/cenexin affects optical refraction and reduces the fluorescent intensity. Thus, the fluorescence intensity when the objective lens is at a higher position is observed as lower than the true intensity. We analysed intensity decay using 500‐nm fluorescent beads (Life Technologies) at various objective heights (Fig. S1). Fluorescent beads diluted to 0.02% solids in PBS were put between coverslips. The beads that adhered to the coverslip were captured using a motorised z‐stage with a built‐in encoder of an Olympus IX81, monitored by Micro‐Manager 34, 35. We set the z‐step of the objective lens to be 0.1 μm. The precise positions of the beads were detected with ‘MetroloJ’ 36, a plug‐in software package for Fiji. Finally, we fitted the intensity as a function of height using an exponential equation by employing the nls function in r software.

Images of mitotic SH‐SY5Y cells were obtained using an Olympus IX81 controlled with Micro‐Manager. We set the z‐step of the objective lens to be 0.5 μm. To quantify the phosphorylation level of Tp1479, three‐dimensional images (after the intensity correction described above) were projected onto two‐dimensional images using Fiji 37. We then manually set an ROI that enclosed a mitotic cell and measured the mean intensity of each ROI. We then calculated the relative intensity, which was normalised by the mean of the measured intensity of the control.

To quantify Wnt3a adsorption on collagen, the background was subtracted from the fluorescent signal. After subtraction, the relative intensity was normalised by the mean intensity of the control.

### Cell culturing in the device

A 150‐μg·mL^−1^ collagen‐coated (Cellmatrix Type IV, Nitta Gelatin, Osaka, Japan) glass bottom dish was washed with ultrapure deionised water once and dried under a stream of nitrogen. The cell culture device was sterilised by autoclaving (20 min at 120 °C). The sterilised device was connected to a vacuum pump (DAP‐15, ULVAC KIKO, Miyazaki, Japan) and placed on the collagen‐coated dish. To prevent the formation of air bubbles in the microchannel, the device was filled with PBS before injecting the culture medium. Inlet tubing was assembled using a 3‐way stopcock (TS‐TR2A, Terumo, Tokyo, Japan), a luer fitting (VRF106, ISIS, Osaka, Japan), silicone tubing (TGK, Tokyo, Japan), a 19‐gauge needle (Terumo) and polytetrafluoroethylene tubing (F8001, FLON INDUSTRY, Tokyo, Japan). The inlet of the device was connected to the inlet tubing and injected with a Wnt3a‐containing medium (Wnt3a‐CM; 100 or 30 ng·mL^−1^). After filling the device with Wnt3a‐CM, the opposite inlet was connected to the inlet tubing and injected with only culture medium. Outlet tubing, which consisted of a 19‐gauge needle and polytetrafluoroethylene tubing, was connected to the outlet port of the device.

The 3‐way stopcock of the inlet tubing was set so as not to wash out the cells from the cell culturing channel by accident. A synchronised cell suspension (2 × 10^6^ cells·mL^−1^; counted by a haemocytometer) was loaded into the cell inlet. After loading the cells, the cell inlet and outlet were plugged to prevent contamination. The device and tubing were placed in a 5% CO_2_ stage top incubator (INUG2‐ONICS, TOKAI HIT, Shizuoka, Japan) at 37 °C and with a micro‐syringe pump (Nexus 3000, Chemyx, Stafford, TX, USA). After setting, the 3‐way stopcock of the inlet tubing was turned, and the flow rate was set at 10.0 μL·min^−1^ for 5 min to form an immediate concentration gradient (Fig. S2). After 5 min, the adhered cells were grown at a flow rate of 2.0 μL·min^−1^ for 12 h.

The angle of the pole‐to‐pole axis determined by the ODF2/cenexin distribution was measured using Fiji. The rose diagram was drawn using a ‘circular’ package installed in R.

## Results

### Development and evaluation of the microfluidic device

**Figure 2:**
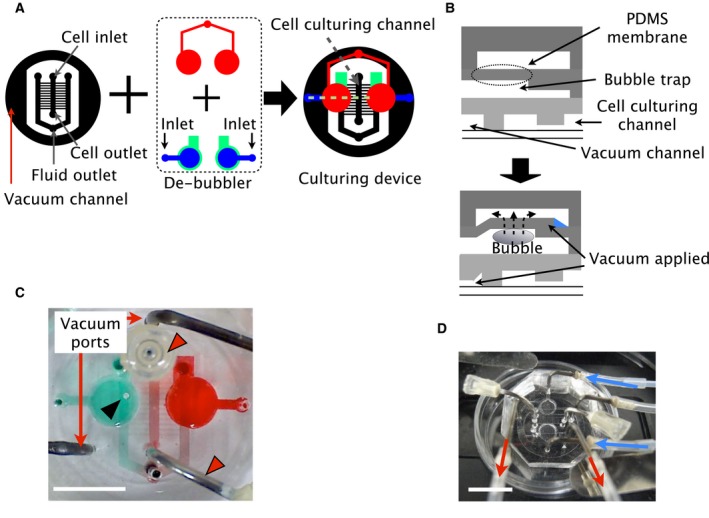
Design of the microfluidic device. (A) Diagram of the microfluidic device. The device has three layers: the pneumatic layer, the fluidic layer and the culturing layer. The culturing layer has a vacuum channel to reversibly bond the PDMS and glass surfaces. The de‐bubbler is designed to remove small bubbles. (B) Diagram of a cross section of the device at the dashed green line in A. The thin PDMS membrane is deformed by applying a vacuum that traps small bubbles. The bubbles can then pass through the PDMS membrane and be removed. The red, blue and grey depict the pneumatic layer, the fluidic layer and the culturing layer, respectively. (C) Actual device. Red triangles indicate lids on the cell inlet and outlet to prevent contamination during cell culturing. The black triangle indicates a bubble trapped in the circular bubble trap. Scale bar = 10 mm. (D) The device set‐up for cell culturing. The blue arrows indicate the stream of the culture medium. The red arrows indicate the stream of the vacuum for the reversible bond between the device and the glass substrate. Scale bar = 10 mm.

We developed a microfluidic device for stimulating SH‐SY5Y cells with a concentration gradient of Wnt3a. To solve the issue of bubbles invading the microchannel, we built bubble traps and a pneumatic de‐bubbler into our device (Figs 2A,B and S3) 26. To reduce unexpected protein adsorption on the PDMS surface, we coated the surface of the microchannel in the device with MPC polymer (Fig. S4) 27.

Figure 2C,D shows the developed device and set‐up on a microscope. The design of the concentration gradient generated in our study was based on previous research 38, 39. We modified the number of outlets from two to one because multiple outlets created a pressure difference that induced an unexpected stream in the culturing channel.

**Figure 3:**
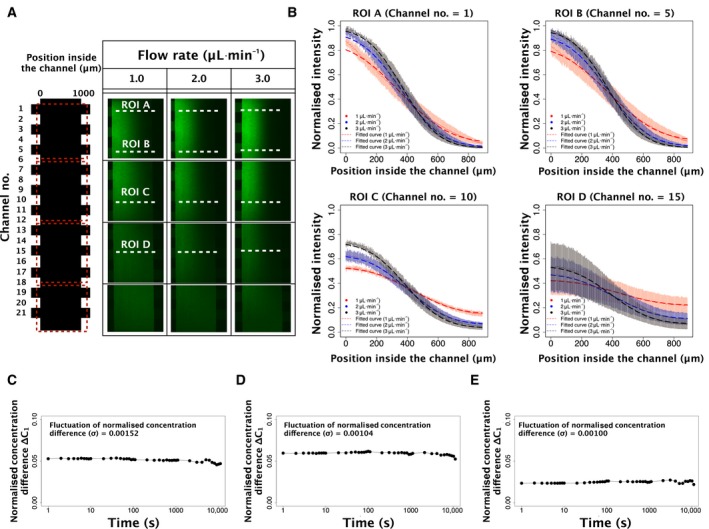
Evaluation of the concentration gradient in the device. (A) Confocal fluorescence images in the culturing channel at each flow rate. The optical slice thickness was approximately 60 μm. Green fluorescence corresponds with the concentration of FITC molecules. (B) The relative intensity of FITC‐dextran is dependent on the position at each ROI in A. Dots indicate experimental data. Dashed lines indicate fitted results using Fick's Law. Error bars show the standard deviation of the normalised intensity. (C), (D) and (E) Stability of the concentration gradient at channel no. 1 over time at a flow rate of 2 μL·min^−1^. Each graph indicates the normalised concentration difference at a specific distance from the microgrooves (C: 200, D: 400 μm and E: 600 μm). The concentration was measured 10 times at each interval of 1, 10, 100 and 1000 s. *x*‐axis: time [s], *y*‐axis: normalised concentration difference at each position indicated above in the cell culturing device by the microgrooves. The values shown in the graph are fluctuations of the normalised concentration difference (σ in Eqn (2)).

Next, we evaluated the concentration gradient in the cell culturing channel at each flow rate using FITC‐dextran which has a molecular weight similar to Wnt3a (Wnt3a: 38 kDa, FITC‐dextran: 40 kDa; Fig. 3A) 38. Because the molecular weights are close, the number of Wnt3a molecules in a 100 ng·mL^−1^ solution approximates the number of FITC molecules in 100 ng·mL^−1^ FITC‐dextran medium. Thus, we estimated that the spatial distribution of the fluorescence of FITC‐dextran mimics the spatial distribution of Wnt3a in the culturing channel.

The concentration gradient was fitted by Fick's Law (Eqn (1)) 28, 29 at four positions in the culturing channel (at channel no. 1 (=ROI A), channel no. 5 (=ROI B), channel no. 10 (=ROI C) and channel no. 15 (=ROI D)). We found that the concentration gradient was fitted well by the equation (Figs 3B and S5A,B). Additionally, we found that the difference in concentration between the two ends of a mitotic SH‐SY5Y cell along the axis of the cell culturing channel (∆*C*_2_; Fig. S5C) in the downstream area (no. 16–no. 20) was relatively larger than that along the axis of Wnt3a gradient (*C*_1_; Fig. S5C) in the upstream area (Fig. S5D). We judged ∆*C*_2_ values in the downstream area (no. 16–no. 20) to be too large compared to ∆*C*_1_ values in the downstream area (no. 16–no. 20). Based on these results, we decided to apply the fitting equations for each channel from no. 1 to no. 15 only. And we analysed the cells in the area (no. 1–no. 15).

As the last step of evaluating our device, we confirmed that the concentration gradient in the cell culturing channel was temporally stable. We observed the normalised concentration difference (∆*C*_1_, Fig. S5C) at channel no. 1, where the residual sum of squares (RSS) against the fitting equation showed the largest value (Fig. S5B). The results showed that the fluctuations (σ) of ∆*C*_1_ were 10 times smaller than the estimated ∆*C*_1_ in various timescales (seconds ~ hours, Fig. 3C–E, the σ values are indicated in each graph as an annotation). The result indicates that the fluctuation of the concentration gradient by unbalanced pressure drops originated from the device structure was small comparing to the concentration gradient which we need for our analyses. Thus, we concluded that influence of the fluctuation could be ignored in our research. Based on this result, we confirmed that the concentration gradient in the cell culturing channel was temporally stable for at least 1–1000 s.

### The concentration gradient of Wnt3a orients the pole‐to‐pole axis

We investigated whether the Wnt3a concentration gradient can produce an asymmetric distribution of ODF2/cenexin in SH‐SY5Y cells during mitosis.

First, we confirmed whether Wnt3a treatment activated Wnt signalling in SH‐SY5Y cells. The LRP6 co‐receptor is one of the most upstream molecules of Wnt signalling. When a cell is stimulated by Wnt, LRP6 is activated by phosphorylation at T1479. If the asymmetric distribution of ODF2/cenexin is regulated by Wnt signalling, this event should be observed following the elevation of phosphorylated T1479 (Tp1479) LRP6 levels in a cell. We confirmed that LRP6 Tp1479 was increased by Wnt3a treatment to approximately 1.5 times that of control cells (Fig. S6). This result confirms that Wnt3a treatment activated Wnt signalling in the SH‐SY5Y cells.

**Figure 4:**
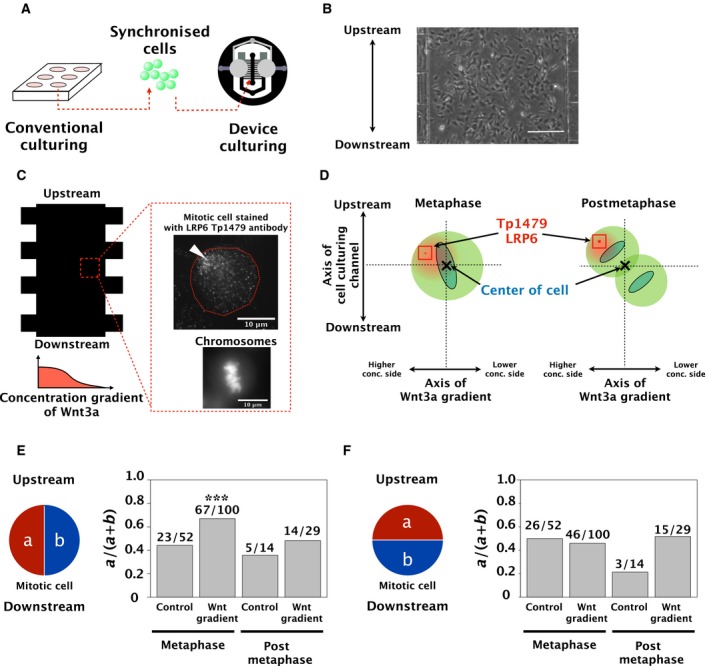
Localisation of phosphorylated LRP6 T1479 stimulated by a Wnt3a concentration gradient during mitosis. (A) Diagram of the experiment. Cells synchronised by a double thymidine block were injected into the culturing channel and cultured for 12 h at a flow rate of 2 μL·min^−1^. (B) Phase‐contrast microscopic image. Cells in the culturing channel were captured after 10 h culturing. Scale bar = 250 μm. The upper side is upstream of the culturing layer. Wnt3a diffuses from the left side of the channel. (C) Immunofluorescent staining of LRP6 Tp1479 during metaphase. Immunostained LRP6 Tp1479 was localised to the higher concentration regions of Wnt3a. The top and bottom sections of the image show upstream and downstream of the culturing channel, respectively. The upper right image shows aligned chromosomes stained by Hoechst 333432. The white triangle indicates phosphorylated LRP6 T1479. (D) Postmetaphase includes anaphase and telophase. The red depicts LRP6 Tp1479, and the blue depicts chromosomes. The ‘X’ shows the centre of a cell; coordinates were relative to the centroid of the chromosomes as the origin. (E) LRP6 Tp1479 at metaphase was localised to the higher concentration side of Wnt3a. The probability (*a*/(*a *+ *b*), *y*‐axis) is the value obtained by dividing the number of cells in which LRP6 Tp1479 was localised at the higher concentration side by the total number of cells. We used a two‐sided, exact binomial test to judge if there was bias in each sample. ***P < 0.001. (F) There was no bias in the localisation of LRP6 Tp1479 in the direction of the flow of medium containing Wnt3a during metaphase. The probability (*a*/(*a* + *b*), *y*‐axis) is the value obtained by dividing the number of cells in which LRP6 Tp1479 was localised to the side of upstream by the total number of cells. We used the two‐sided, exact binomial test to judge whether there is a bias in each sample, and no significant difference was found.

We synchronised SH‐SY5Y cells with thymidine to efficiently identify mitotic SH‐SY5Y cells in the device (Fig. 4A). We then cultured and stimulated the cells with a Wnt3a concentration gradient at a flow rate of 2 μL·min^−1^ for 12 h (Fig. 4B). Under these conditions, we confirmed whether the Wnt3a concentration gradient in the device could induce biased‐activation of Wnt3a signalling at the higher concentration side in stimulated mitotic SH‐SY5Y cells, by checking the localisation of LRP6 Tp1479. To define the activated area in a cell, we set an 11 × 11 [pixel] window for measurement and analysed the activation level of LRP6 (Fig. 4C,D). We defined the centre coordinates of the 11 × 11 [pixel] window which had the highest signal of LRP6 Tp1479, as the activated position (Fig. 4D, indicated as a red square and a dot). At the same time, we defined the centroid of the chromosomes as the centre of a cell during metaphase. We defined the centre of a line across two centroids of divided chromosomes as the centre of a cell in postmetaphase cells, which include anaphase and telophase cells (Fig. 4D, indicated as ‘*x*’).

Figure 4E shows that the Wnt3a concentration gradient succeeded in inducing biased‐activation of Wnt3a signalling at the higher concentration side of Wnt3a in stimulated cells in metaphase. At the same time, a significant bias in the LRP6 Tp1479 distribution towards the direction of the media flow was not observed (Fig. 4F).Figure S7 shows all the plots of each experimental set for Fig. 4E,F to show the distribution of these data. These data suggest that the bias in the LRP6 Tp1479 distribution towards the Wnt3a concentration gradient is induced by Wnt signalling and does not occur randomly.

**Figure 5:**
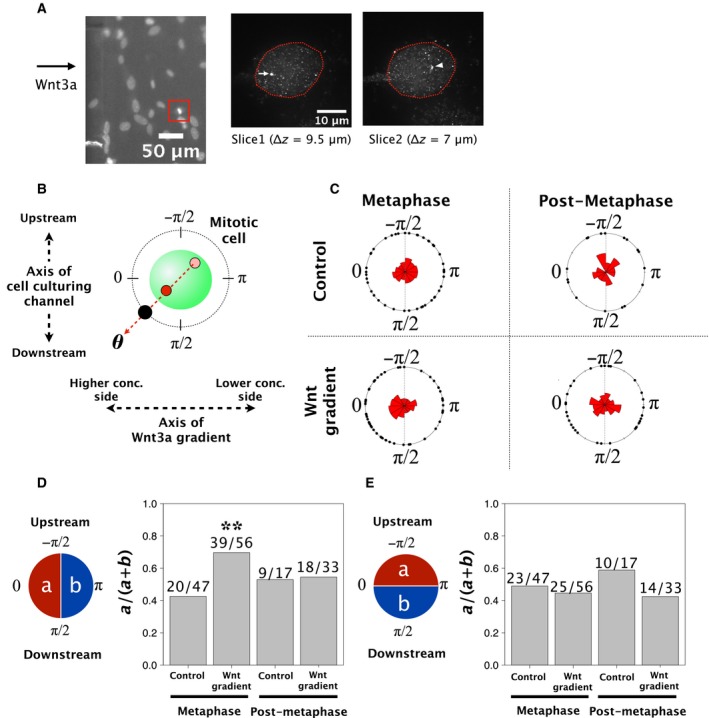
Orientation of the pole‐to‐pole axis during mitosis under a Wnt3a concentration gradient. (A) The leftmost image shows chromosomes stained with Hoechst in the cell culturing channel after immunostaining (× 10 objective lens). The two confocal immunofluorescent images on the right show ODF2/cenexin staining in the metaphase cell in the red frame of the leftmost image (× 100 objective lens). The two immunofluorescent images were acquired at different z‐positions. The white arrow indicates the localisation of ODF2/cenexin with high intensity, and the white triangle indicates the localisation of ODF2/cenexin with low intensity. (B) Diagram of the measurement angle of the pole‐to‐pole axis. The red circle indicates a higher intensity of ODF2/cenexin, and the pink circle indicates a lower intensity of ODF2/cenexin. The upstream direction of the culturing channel was an angle of ‐π/2, and the direction towards the wall with microgrooves for draining Wnt3a‐CM had an angle of 0. (C) Angle histogram of the pole‐to‐pole axis under each mitotic phase and condition. ‘Control’ means that culture medium was injected into both inlets. ‘Wnt gradient’ means that Wnt3a‐CM and culture medium were injected separately into the two inlets. (D) The probability that the orientation of the pole‐to‐pole axis is biased towards the higher concentration side of Wnt3a. ODF2/cenexin localised to the side with the highest Wnt3a concentration was statistically significant during metaphase. The value of the axis towards and against 0 degrees was counted (a: ‐π/2 ≦ θ < π/2, and b: ‐π ≦ θ < ‐π/2 and π/2 ≦ θ < π). We used a two‐sided, exact binomial test. **P < 0.01. (E) The probability of the orientation of the pole‐to‐pole axis towards the upstream side. There was no significant bias between the upstream and downstream sides. The value towards and against ‐π/2 was counted (a: ‐π ≦ θ < 0, and b: 0 ≦ θ < π).

To further analyse the quantitative effect of the Wnt3a concentration gradient, we measured the angle between the pole‐to‐pole axis and the axis of the Wnt3a gradient among the mitotic cells. The leftmost panel in Fig. 5A shows the cells after immunostaining with anti‐ODF2/cenexin in the cell culturing channel. We confirmed that we could detect ODF2/cenexin based on differences in the fluorescence intensity of the protein (Fig. 5A, middle and right panels).

Figure 5B shows the definition of the two axes and the angle measured in our analyses. We also divided our analysis into two mitotic phases: metaphase or postmetaphase. Figure 5C shows the distribution of the pole‐to‐pole axis angles in four different conditions. These results demonstrate that the Wnt3a gradient orients the pole‐to‐pole axis towards the higher concentration side of Wnt3a in metaphase (39/56, *P* = 0.0046, Fig. 5D). In contrast, there was no significant bias in the orientation of the pole‐to‐pole axis towards the direction of the media flow, independent of any phase of mitosis or the presence of a Wnt3a concentration gradient (Fig. 5D,E). All the ratio plots of each experimental set were in Fig. S8. These results support the conclusion that a Wnt3a gradient can orient the pole‐to‐pole axis during metaphase.

Because Wnt3a is highly adsorptive to extracellular matrices (Fig. S9A,B) 40, we examined whether the Wnt3a concentration gradient formed and was maintained on the collagen surface during cell culture. We measured the extent of the heterogeneous distribution of the adsorbed Wnt3a inside the culturing channel by observing the difference in fluorescence values between the Wnt3a‐CM area (Area 1) and the control area (Area 2; Fig. S9C,D). SH‐SY5Y cells were cultured on the collagen‐coated substrate where a heterogeneous distribution of Wnt3a was formed in medium without Wnt3a supplementation. We confirmed that there was no significant bias in the orientation of the pole‐to‐pole axis despite this heterogeneous Wnt3a distribution on the extracellular matrices (Fig. S9E,F).

### High sensitivity to the Wnt3a gradient in the orientation of the pole‐to‐pole axis during mitosis

We quantitatively evaluated how much of a Wnt3a concentration gradient is needed to orient the pole‐to‐pole axis towards the higher concentration side of Wnt3a. To calculate the gradients of Wnt3a between the two ends of the mitotic SH‐SY5Y cells (∆*C*) at different positions in the device, we applied Eqn (1). We assumed that the gradients were linear within the diameter of a mitotic SH‐SY5Y cell as it is approximately 100 times smaller than the width of the microchannel.

**Figure 6:**
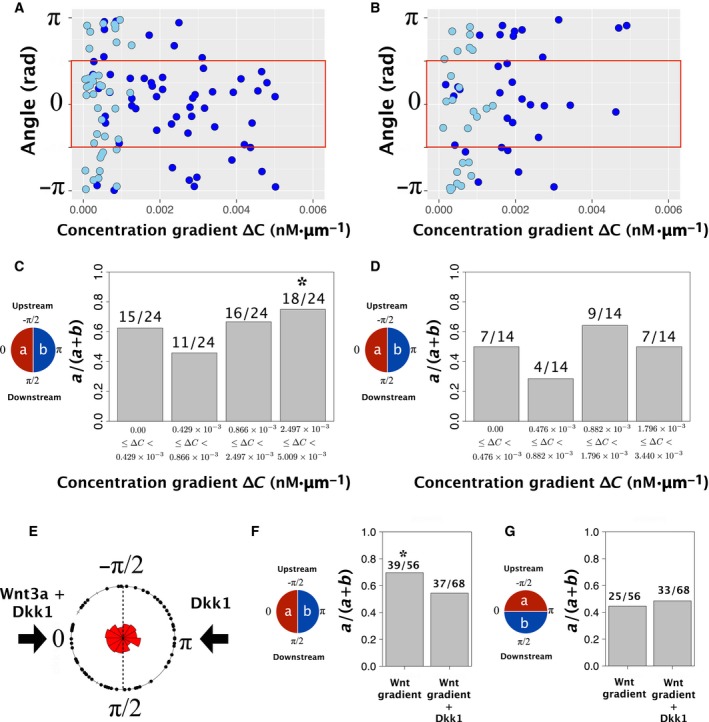
Relationship between the angle of the pole‐to‐pole axis and the Wnt3a concentration gradient. (A) Plot of the angle of the pole‐to‐pole axis at each concentration gradient of Wnt3a (∆*C*) during (A) metaphase and (B) postmetaphase. The rectangular area outlined in red indicates the angle towards microgrooves which drain Wnt3a‐CM. Light blue dots indicate 30 ng·mL^−1^ Wnt3a. Dark blue dots indicate 100 ng·mL^−1^ Wnt3a. (C) The biased ratio of the pole‐to‐pole axis in cells in metaphase exposed to the high Wnt3a‐concentration side. The number of concentration gradient ranges was divided into four. The number of cells in each concentration range was the same (e.g. if the total number of cells equalled 40, there would be 10 cells in each of the four concentration ranges). We performed a two‐sided, exact binomial test. *P < 0.05. (D) The biased ratio of the pole‐to‐pole axis to the high Wnt3a concentration side in postmetaphase. There were four concentration gradient ranges. We performed a two‐sided, exact binomial test and found no significant difference. (E) Angle histogram of the pole‐to‐pole axis during metaphase when Wnt3a and Dkk1 were injected into the device. The angle of the pole‐to‐pole axis was random when Dkk1 was added to the cell culturing environment. (F) The biased ratio of the pole‐to‐pole axis to the high Wnt3a concentration side with the addition of Dkk1 in metaphase. There was a significant difference without Dkk1 (left bar); however, there was no significant difference when Dkk1 was added (right bar). The statistical analysis was a two‐sided, exact binomial test. *P < 0.05. The *P*‐value for the condition of Dkk1 addition during metaphase was 0.544. (G) The biased ratio of the pole‐to‐pole axis to medium flow direction when Dkk1 was added during metaphase. We performed a two‐sided, exact binomial test, and there was no significant difference independent of Dkk1 addition.

We performed further experiments stimulating SH‐SY5Y cells with a low concentration of Wnt3a (30 ng·mL^−1^) to analyse the relationship between ∆*C* and the angle of the pole‐to‐pole axis at each mitotic phase (Fig. 6A,B). We divided the cell population into four groups depending on the concentration gradient of Wnt3a; 0.00–0.429, 0.429–0.866, 0.866–2.497 and 2.497–5.009 × 10^−3^ [nm·μm^−1^]. We performed a binomial test to determine whether the pole‐to‐pole axis orientation was biased towards higher concentrations of Wnt3a. The angle of the pole‐to‐pole axis in cells undergoing metaphase converged at Wnt3a concentrations of 2.497–5.009 × 10^−3^ [nm·μm^−1^] (Fig. 6C). In all other conditions, including smaller concentration gradients in metaphase cells and all concentration gradients in postmetaphase cells, there was no significant bias in pole‐to‐pole axis orientation (Fig. 6C,D). This result indicates that the minimum sensitivity threshold of SH‐SY5Y cells in our experiment is a Wnt3a concentration of 2.5 × 10^−3^ nm·μm^−1^. All the ratio plots in each concentration gradient group were in Fig. S10. These results support the conclusion that the orientation of the pole‐to‐pole axis depends on concentration gradient of Wnt3a.

We also performed a control analysis without supplying Wnt3a and tested whether other factors (e.g. media flow) in the culturing device may affect the above results. We confirmed that there was no significant effect on pole‐to‐pole axis determination without Wnt3a present (Fig. S11).

Finally, to determine the sensitivity to the Wnt3a gradient, we injected a Wnt3a antagonist Dkk1, which binds to the same receptors as Wnt3a, into both fluidic inlets. The angle of the pole‐to‐pole axis was randomised with Dkk1 (Fig. 6E). A two‐sided, exact binomial test indicated that the pole‐to‐pole axis was biased towards the Wnt3a gradient only in the absence of Dkk1 (Fig. 6F,G). All the ratio plots of each experimental set were in Fig. S12.

These results suggest that the threshold for sensing a gradient of Wnt3a by SH‐SY5Y cells is 2.5 × 10^−3^ nm·μm^−1^ and that a steeper gradient contributes to determining the pole‐to‐pole axis during cell division.

## Discussion

In this paper, we examined the important hypothesis that the spatial signalling of Wnt3a can produce asymmetric cellular dynamics during mitosis. We particularly focused on identifying the quantitative threshold of the Wnt3a concentration gradient that is required to determine the pole‐to‐pole axis. This report provides the first evidence clarifying this threshold and demonstrates the role of the Wnt3a concentration gradient in determining the division axis. Evidence from the microfluidic experiment in Fig. 5 shows that the higher accumulation of ODF2/cenexin at the centriole in metaphase was biased towards higher concentrations of Wnt3a. In zebrafish gastrulation, the rotation of the mitotic spindle is directed by the animal–vegetal axis until anaphase B 41. This directed mitotic rotation is triggered by Wnt signalling 41.

Another supportive example for the role of Wnt signals in ES cells is suggested by our research. Until the end of cell division, a grandmother centriole associates with the distal/subdistal appendage protein Ninein, which requires accumulation of ODF2/cenexin on the mother centriole 42. A previous study demonstrated that the grandmother centriole was inherited to the daughter cell that was attached to Wnt3a‐coated beads in 78% of ES cells 7. By comparing Wnt3a signalling in various species, in the light of our quantitative findings, we highlight the significance of evolutionarily conserved polarised signalling mechanisms.

The distribution of a grandmother centriole is closely related to the axis of asymmetric cell division in *Drosophila* cells, neural stem cells and ES cells 7, 23, 24. Based on this observation, ODF2/cenexin is likely to be one of the factors which define the cell division axis. Further investigation of the function of this protein may help to understand how asymmetric cell division is coordinated in neuroblastoma and the other cells.

How the distribution of Wnt3a *in vivo* contributes to the processes of development via controlling the pole‐to‐pole axis of cell division is a point of contention. Wnt proteins are hydrophobic; during the processes of development, they are tethered to the plasma membrane and extracellular matrices 18, 40. As a result, the concentration gradient of Wnt3a is supposedly formed on the plasma membrane and extracellular matrices.

Our analyses indicated that mitotic SH‐SY5Y cells have high sensitivity to the concentration gradient of Wnt3a (Fig. 6). High sensitivity to the gradient of signalling molecules has been reported in the study of chemotaxis in *Dictyostelium* cells 43. Chemotaxis requires the formation of a polarity motif and a sensing motif for extrinsic signalling cues. During self‐organisation of polarity, the domain of high phosphatidylinositol (3,4,5)‐trisphosphate concentration, which contributes to actin polymerisation was spontaneously generated in the cell membrane region, independently from the distribution of extrinsic cues 44, 45, 46. In contrast, the sensing of extrinsic cues was thought to be contributed to by the sensing motif, which is independent of the polarity formation motif. Recently, several theoretical models of polarity formation and sensing motif formation have been reported, including the local excitation/global inhibition (LEGI) model and models based on LEGI 46, 47, 48. SH‐SY5Y cells in metaphase may utilise a similar mechanism to sense small concentration difference.

The pole‐to‐pole axis position is pulled and moved by astral microtubules that originate at the pole and reach the cell cortex 49. We propose that the pulling forces acting on microtubules could be combined with a sensing mechanism for Wnt3a. Elucidating these mechanisms would require high‐speed imaging technologies such as those we developed using electrically tunable lenses 50. Given such exquisitely sensitive mechanisms for sensing extrinsic cues as those reported in *Dictyostelium* cells, the concentration gradient of Wnt3a in the extracellular matrix may function as the extrinsic cues in the present case, with the LRP6 co‐receptor working as the sensing mechanism.

The advantage of our device is that it can vary the cell culturing conditions by controlling the flow rate. Additionally, the produced concentration gradient of solutes is stable (Fig. 3). By using two independent inlets to produce a stable gradient, this device can combine the gradient pattern of multiple stimulation molecules.

As we have shown in this paper, our device has the potential to contribute to uncovering the mechanisms of asymmetric cell division and the self‐organisation of polarity by mimicking the polarised spatial pattern of developmental secretory protein. Using this device, we have established for the first time that a Wnt3a concentration gradient is sufficient to determine the pole‐to‐pole axis orientation, and we established the sensitivity threshold of cells to Wnt3a in spindle axis positioning during mitosis. Quantitative study of extracellular cues such as those indicated in this paper will become increasingly important to answer how small signalling gradients govern complex downstream mechanical processes.

## Conflict of interest

The authors declare no conflict of interest.

## Author contributions

TH designed and performed all critical experiments, theoretical analyses and statistical tests in this paper, and wrote most of the manuscript. HK, NM, YM, NH and AF cooperated in the planning of this project, and NH conducted the whole research project in detail, in constant discussion with AF. YN contributed to the micromanager setting and 3D imaging. TY contributed to the statistical tests. RT helped to manage the cell culturing.

## Supporting information


**Fig. S1.** Calibration of intensity according to the objective height. (A)Diagram of the z‐calibration experiment. The sample was prepared by overlaying two coverslips. Fluorescent beads adsorbed on the coverslip were observed using a × 100 objective lens. The z‐position of the objective lens, which focused on the beads on the coverslip closest to the objective lens, was defined as the reference height (z_0_). The difference between the z‐position of the objective lens which was then focused on the beads just under the furthest coverslip from the objective lens and the reference height was defined as ∆*z*. (B) The relationship between ∆*z* and the relative intensity. We set the average intensity of the beads at the reference height to 1.0. We hypothesised that the reduction in intensity was exponential with the offset value, which was defined as the relative intensity at the maximum reduction for the objective lens to touch the coverslip. The parameter values of a, b, and c were 0.645, −0.200 and 0.352, respectively. *P*‐values of parameters were less than 2.0 × 10^−16^. The null hypothesis of this test was that the parameter equals zero.

**Fig. S2.** Perfusion time until stable gradient. (A) Confocal fluorescence images of FITC‐dextran in the cell culturing channel (channel no. 1 ∼ 5). ‘0 min’ shows the timing that the perfusion started. (B), (C), and (D) Fluctuation of the concentration gradient at channel no. 1 over time at a flow rate of 10 μL·min^−1^ from the start of perfusion. Each graph indicates the normalised concentration difference at a specific distance from the microgrooves (C: 200, D: 400, and E: 600 μm). The concentration was measured at the interval of 2 min. *x*‐axis: time [min], *y*‐axis: normalised concentration difference at each position indicated above in the cell culturing device by the microgrooves. Dashed lines indicate the time of 5 min from the start.

**Fig. S3.** Detailed design of the microfluidic cell culture device. Detailed design of the fluidic layer (left), the culturing layer (middle), and the pneumatic layer (right). The upper panel is a magnification of the rectangular area outlined in red in the culturing layer. The lower panel is the cell culture device made by bonding all layers. All units are in millimetres.

**Fig. S4.** Blocking nonspecific protein adsorption on the PDMS surface. (A) Microscopic images of adsorbed BSA‐FITC on the straight microchannel. Nonspecific adsorption of BSA‐FITC on the untreated surface (left) and the MPC polymer dissolved in absolute ethanol‐coated surface (middle). The surface coated with MPC polymer dissolved in a mixture of chloroform and ethanol (right) reduces BSA‐FITC adsorption. Scale bar = 200 μm. (B) Quantification of the adsorption of BSA protein. (C, D) The device, whose channel was coated with MPC polymer dissolved in a mixture of chloroform and ethanol, was fixed with methanol (C), or with PFA (D), and washed with ethanol followed by heat sterilisation. Blocking capacity was retained through several repeated wash and heat cycles. The *y*‐axes of graphs in C and D refer to the relative intensity of BSA‐FITC fluorescence after the indicated number of wash and heat cycles to the intensity after the first cycle.

**Fig. S5.** Profiling of the concentration gradient. (A)Experimental data of normalised intensity (dots) and fitted curve for the data (dashed line) in channel no. 1, at a flow rate of 2 μL·min^−1^. The residual sum of squares (RSS) value was 0.1059. Error bars show the standard deviation of the normalised intensity. (B) The RSS value in each channel. (C) Diagram of the concentration difference between the two ends of a mitotic cell in relation to the axis of the Wnt3a gradient and the axis of the cell culturing channel (∆*C*_1_ and ∆*C*_2_). ∆*C*_1_ is calculated by ${\sqrt{C_{B}\left(x-\frac{d}{2}\right)-C_{B}\left(x+\frac{d}{2}\right)}}^{2}$ and Δ*C*_2_ is calculated by ${\sqrt{C_{A}\left(x\right)-C_{C}\left(x\right)}}^{2}\times d/w$. Functions *C*_A,B,C_ are the concentration gradient equations in (1) for ROI A, B, and C, respectively. Dependent variable *x* is the position of each channel. Parameter *d* is the diameter of the mitotic cell (d = 11 pixels, approximately 30 μm). Parameter *w* is the width of the microgrooves. (D) ∆*C*_1_ (red curve) and ∆*C*_2_ (black curve) in each channel. The horizontal axis indicates the position (μm) across the culturing channel. The vertical axes are the concentration difference of the Wnt3a gradient (left axis: ∆*C*_1_, right axis: ∆*C*_2_). The upper left number shows the channel no. Error bars show the standard deviation of the normalised concentration difference.

**Fig. S6.** LRP6 T1479 activation during mitosis by Wnt3a treatment. (A) Immunofluorescence staining of LRP6 Tp1479 during mitosis. Scale bar = 10 μm. (B) Quantification of the relative phosphorylation level of LRP6 Tp1479 (Control; *n* = 45, Wnt3a; *n* = 46). Mean ± standard deviation of the mean are displayed. A Student t‐test was performed. ***P < 0.001.

**Fig. S7.** All plots of each experimental set for Fig. 4E,F. (A) LRP6 Tp1479 at metaphase was localised to the higher concentration side of Wnt3a. The probability (*a*/(*a* + *b*), *y*‐axis) is the value obtained by dividing the number of cells in which LRP6 Tp1479 was localised at the higher concentration side by the total number of cells in each experiment. (B) The probability (*a*/(*a* + *b*), *y*‐axis) is the value obtained by dividing the number of cells in which LRP6 Tp1479 was localised to the side of upstream by the total number of cells in each experiment.

**Fig. S8.** All plots of each experimental set for Fig. 5D,E. (A) The probability that the orientation of the pole‐to‐pole axis is biased towards the higher concentration side of Wnt3a in each experiment. The value of the axis towards and against 0 degree was counted. Among 6 sets of experiments with metaphase cells in the Wnt gradient, once were less biased towards higher concentration side of Wnt3a (1/3), the other 5 times were biased towards higher concentration side of Wnt3a. (B) The probability of the orientation of the pole‐to‐pole axis towards the upstream side in each experiment. The value towards and against ‐π/2 was counted.

**Fig. S9.** Wnt3a adsorption on collagen. (A)Fluorescent images of collagen‐coated glass and Wnt3a‐adsorbed collagen‐coated glass. The collagen‐coated glass was incubated with either culturing medium only or Wnt3a‐CM. After incubation, Wnt3a adsorption was detected by anti‐Wnt3a antibodies. (B) Quantification of Wnt3a adsorption. The intensity of each experiment was obtained by calculating the average intensity of five fluorescent images. The relative intensity was obtained by dividing each intensity by the average intensity of the control. The individual experiments for each condition were repeated three times. ***P < 0.001: Student t‐test. (C) Diagram of measuring areas in the culturing channel. The relative intensity was measured in the areas near the microgrooves (Area 1 and Area 2). For the control experiment in the culturing channel, culturing medium was perfused from both sides. For the Wnt3a adsorption experiment in the culturing channel, Wnt3a‐CM and culturing medium was perfused from each side of the culturing channel. (D) Profiling the Wnt3a adsorption experiment. Black dots and triangles indicate the average of the relative intensity in Area 1 and Area 2 after 12 h of perfusion at a flow rate of 2 μL·min^−1^. Error bars show the standard deviation of the relative intensity. *P < 0.05, **P < 0.01: Student t‐test. (E) The cells were cultured on Wnt3a adsorbed collagen‐coated glass. The probability of the orientation of the pole‐to‐pole axis towards Area 1 and the upstream side of Wnt3a adsorbed collagen‐coated glass is shown in the right panel. There was no significant bias in the orientation of the pole‐to‐pole axis using a two‐sided, exact binomial test. (F) All plots of each experimental set for (E).

**Fig. S10.** All plots of each experimental set for Fig. 6C,D (A) The biased ratio of the pole‐to‐pole axis in metaphase cells exposed to the high Wnt3a‐concentration side in each experiment. The number of concentration gradient ranges was divided into four. (B) The biased ratio of the pole‐to‐pole axis in postmetaphase cells exposed to the high Wnt3a‐concentration side in each experiment.

**Fig. S11.** Relationship between the angle of the pole‐to‐pole axis and the culturing environment without a Wnt3a concentration gradient. A plot of the angle of the pole‐to‐pole axis without a Wnt3a gradient (∆*C*) during (A) metaphase and (B) postmetaphase in Fig. 5D. We calculated a virtual concentration gradient when Wnt3a‐CM was injected. (C) Probability that the pole‐to‐pole axis orientates towards the microgrooves which drain Wnt3a‐CM during metaphase, and (D) postmetaphase. We performed a two‐sided, exact binomial test and there was no significant difference. (E and F) All plots of each experimental set for (C) and (D).

**Fig. S12.** All plots of each experimental set for Fig. 6F,G. (A) The biased ratio of the pole‐to‐pole axis to the high Wnt3a concentration side with the addition of Dkk1 in metaphase. (B) The biased ratio of the pole‐to‐pole axis to medium flow direction when Dkk1 was added during metaphase.

**Table S1.** Parameters of mould fabrication.
