# Supplementary figures and images for: Quantitative analysis of sensitivity to a Wnt3a gradient in determination of the pole‐to‐pole axis of mitotic cells by using a microfluidic device
Source: FEBS Open Bio. 2018 Nov 9;8(12):1920–35. doi: 10.1002/2211-5463.12525 (PMC6275273; doi:10.1002/2211-5463.12525)

**A**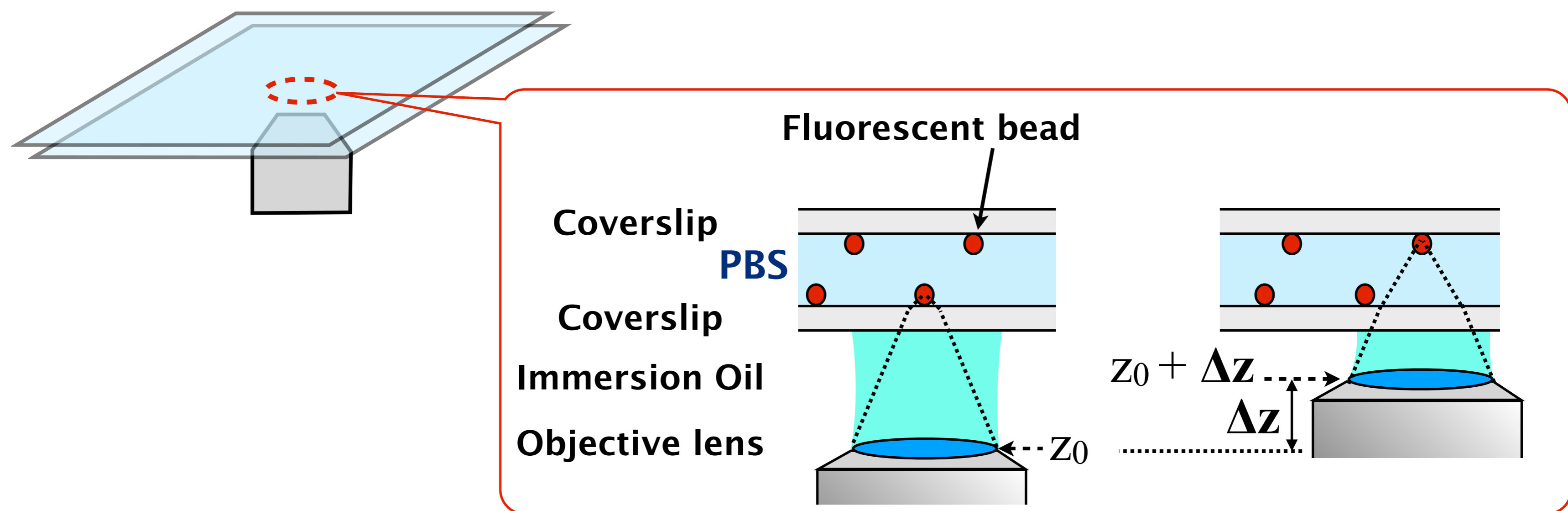**B**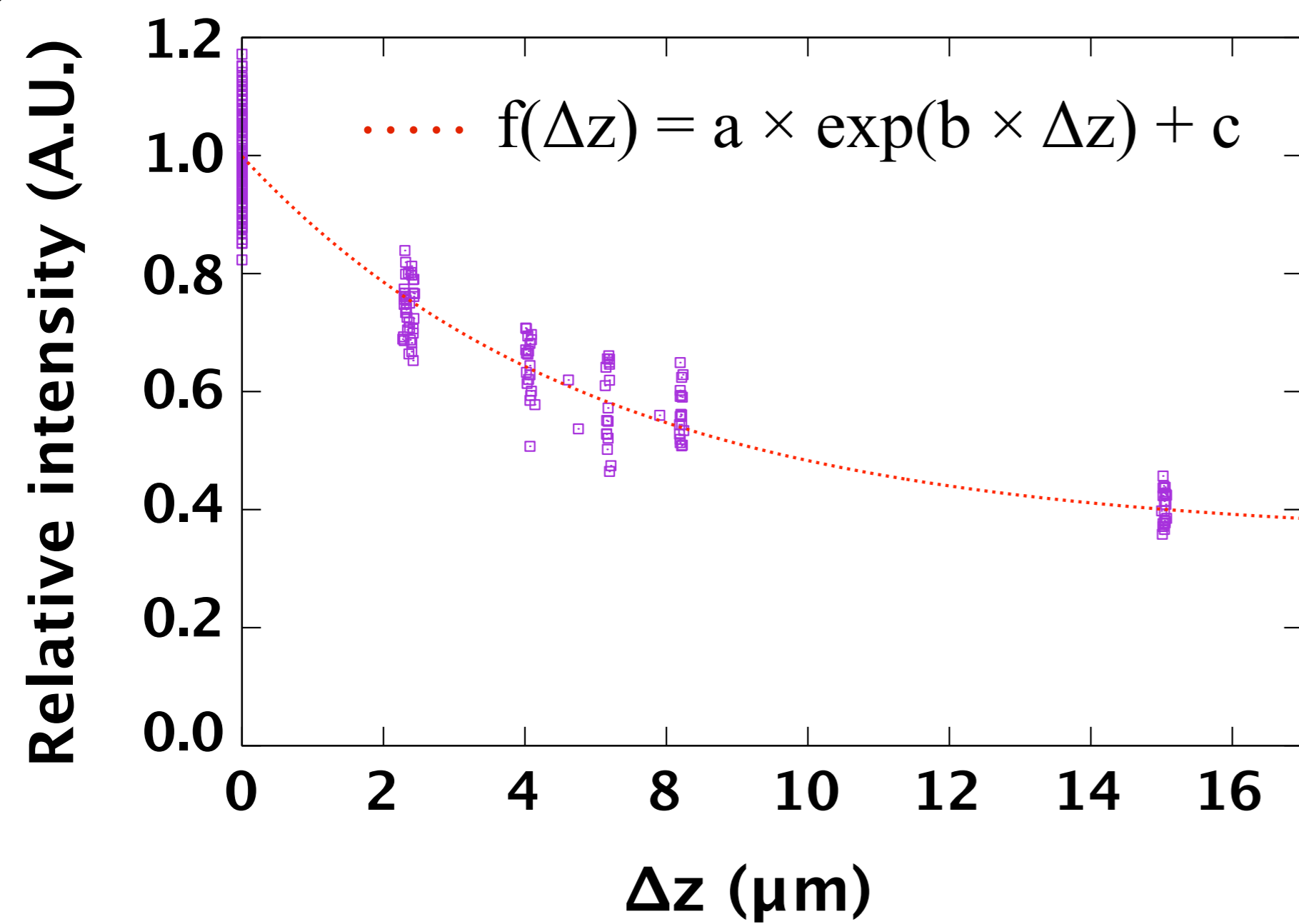

Supplement: Supplementary file 1 — Fig. S1. Calibration of intensity according to the objective height. (A)Diagram of the z‐calibration experiment. The sample was prepared by overlaying two coverslips. Fluorescent beads adsorbed on the coverslip were observed using a × 100 objective lens. The z‐position of the objective lens, which focused on the beads on the coverslip closest to the objective lens, was defined as the reference height (z0). The difference between the z‐position of the objective lens which was then focused on the beads just under the furthest coverslip from the objective lens and the reference height was defined as ∆z. (B) The relationship between ∆z and the relative intensity. We set the average intensity of the beads at the reference height to 1.0. We hypothesised that the reduction in intensity was exponential with the offset value, which was defined as the relative intensity at the maximum reduction for the objective lens to touch the coverslip. The parameter values of a, b, and c were 0.645, −0.200 and 0.352, respectively. P‐values of parameters were less than 2.0 × 10^−16^. The null hypothesis of this test was that the parameter equals zero. [file FEB4-8-1920-s001.pdf]

**A**

Time: 0 min

4 min

6 min

10 min

20 min

30 min

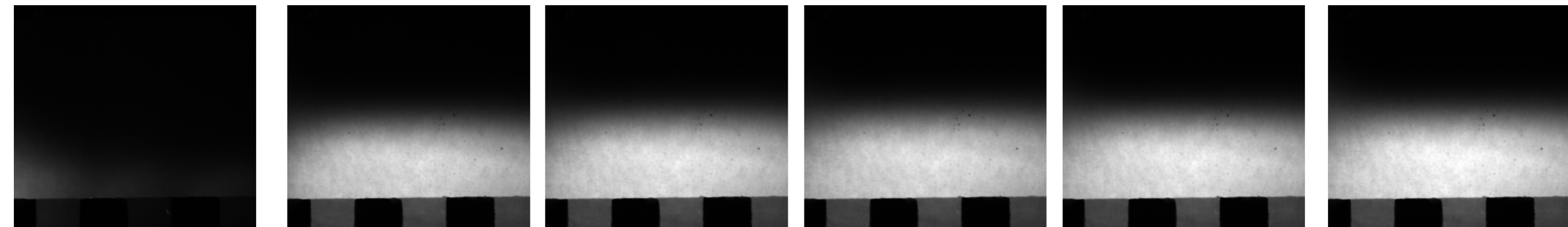**B**Position: 200  $\mu\text{m}$ 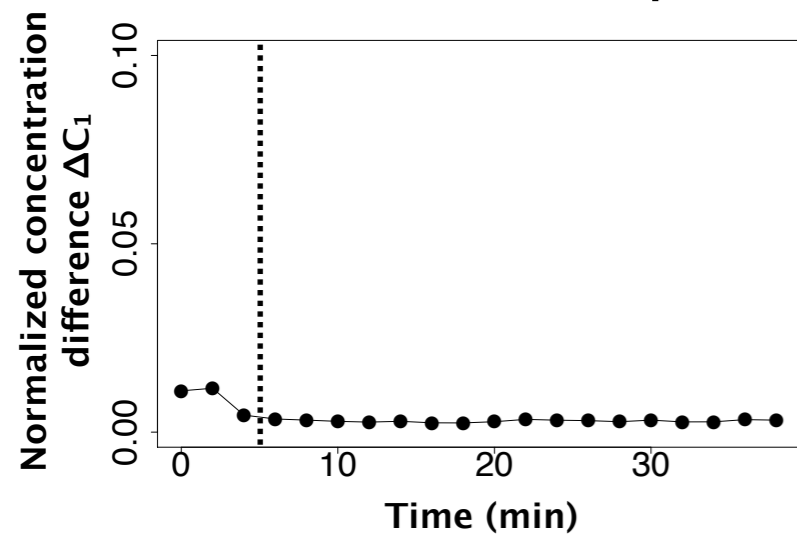**C**Position: 400  $\mu\text{m}$ 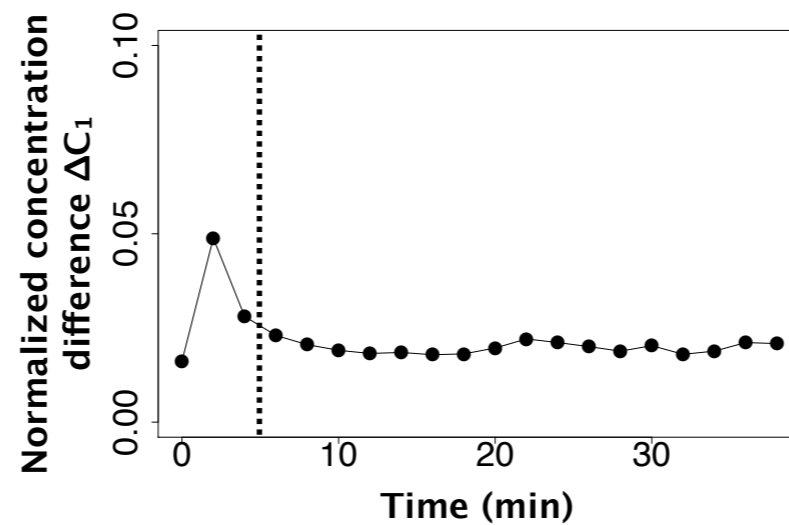**D**Position: 600  $\mu\text{m}$ 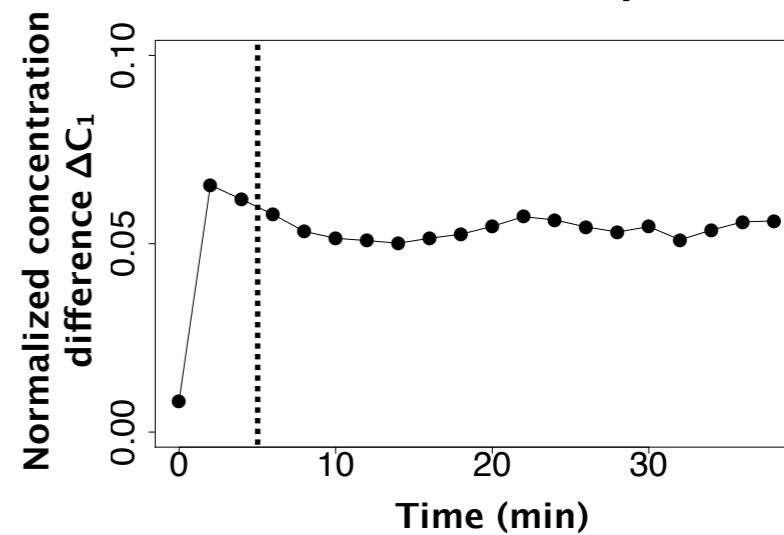

Supplement: Supplementary file 2 — Fig. S2. Perfusion time until stable gradient. (A) Confocal fluorescence images of FITC‐dextran in the cell culturing channel (channel no. 1 ∼ 5). ‘0 min’ shows the timing that the perfusion started. (B), (C), and (D) Fluctuation of the concentration gradient at channel no. 1 over time at a flow rate of 10 μL·min−1 from the start of perfusion. Each graph indicates the normalised concentration difference at a specific distance from the microgrooves (C: 200, D: 400, and E: 600 μm). The concentration was measured at the interval of 2 min. x‐axis: time [min], y‐axis: normalised concentration difference at each position indicated above in the cell culturing device by the microgrooves. Dashed lines indicate the time of 5 min from the start. [file FEB4-8-1920-s002.pdf]

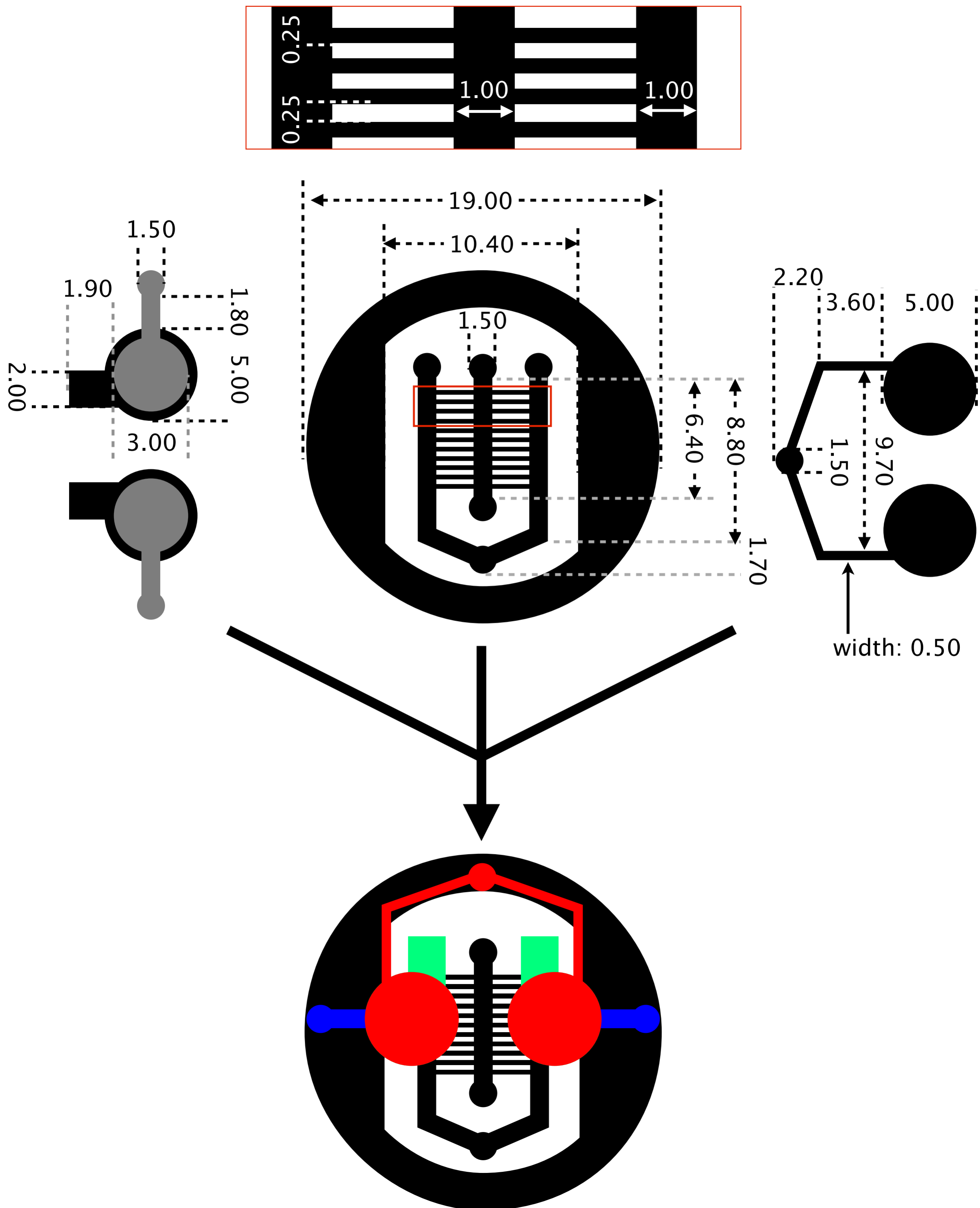

Supplement: Supplementary file 3 — Fig. S3. Detailed design of the microfluidic cell culture device. Detailed design of the fluidic layer (left), the culturing layer (middle), and the pneumatic layer (right). The upper panel is a magnification of the rectangular area outlined in red in the culturing layer. The lower panel is the cell culture device made by bonding all layers. All units are in millimetres. [file FEB4-8-1920-s003.pdf]

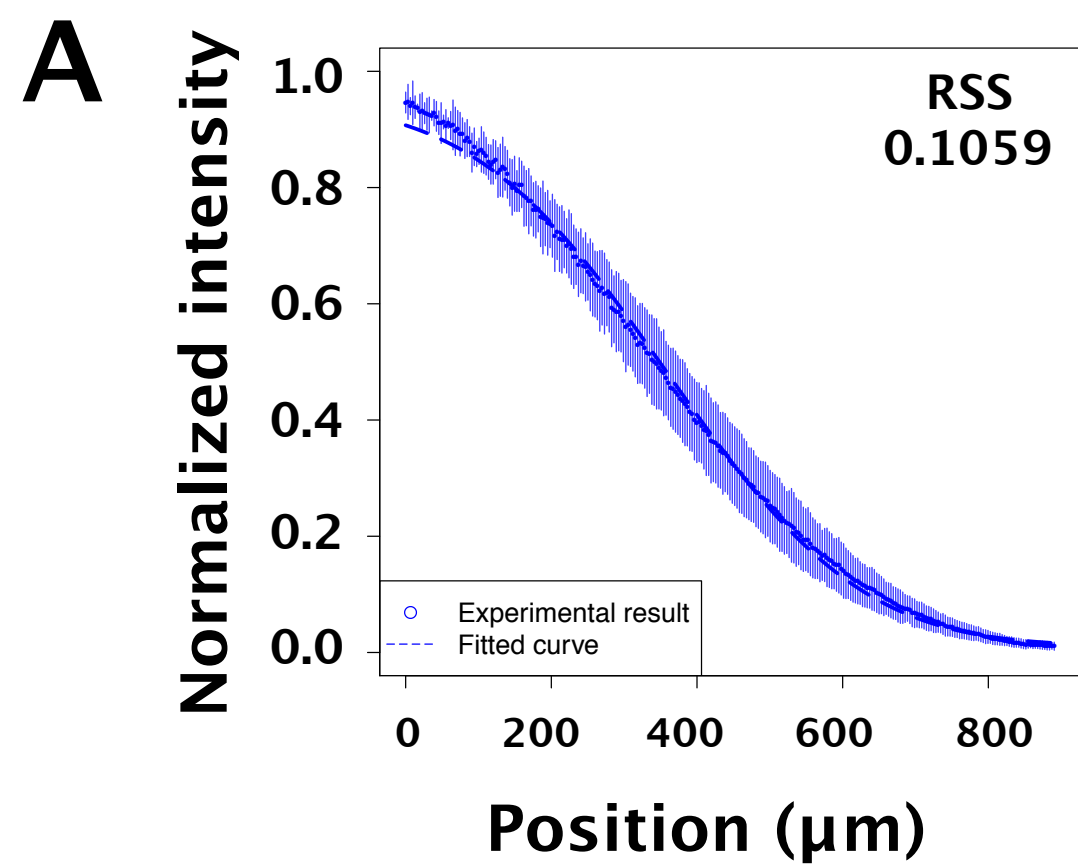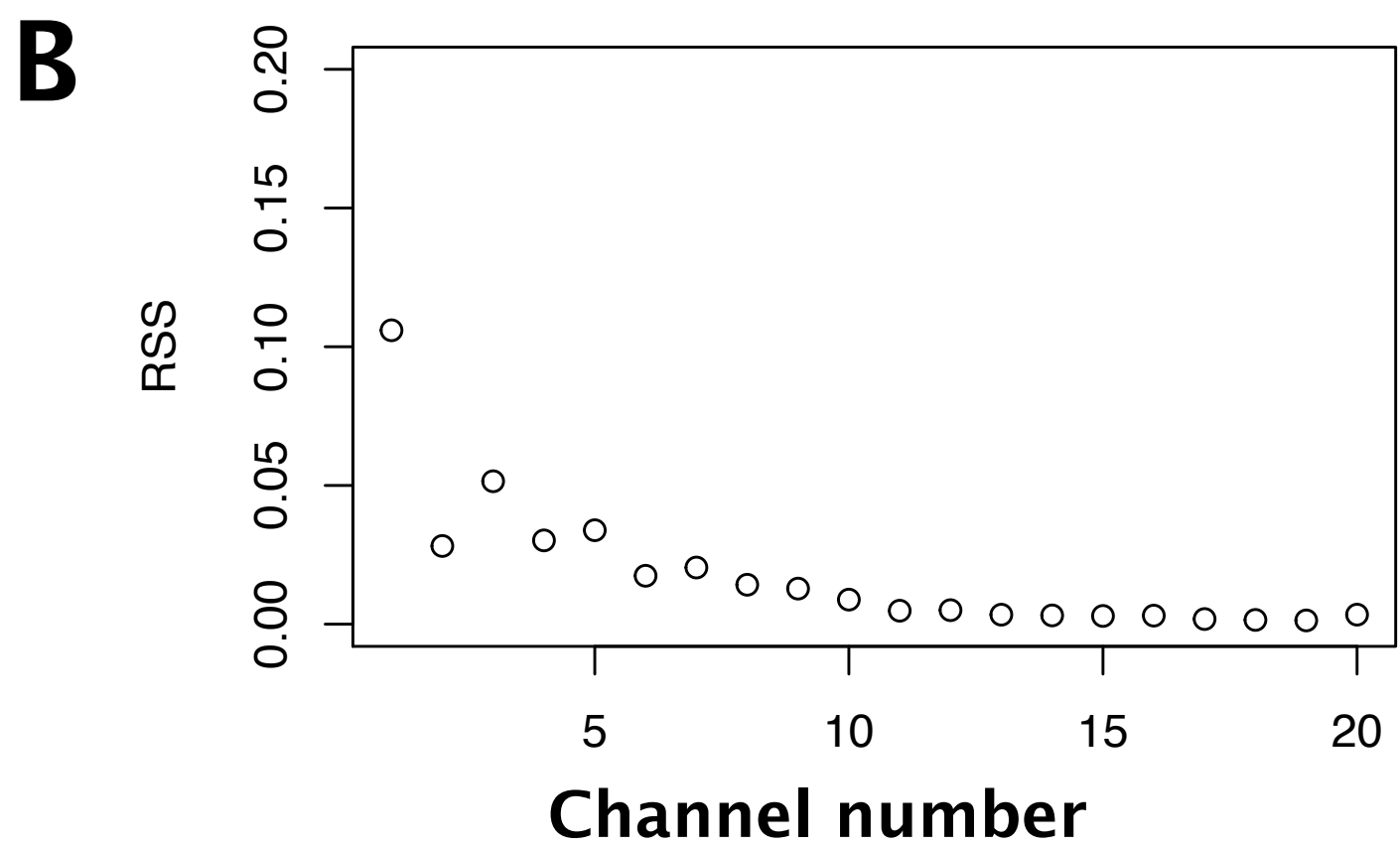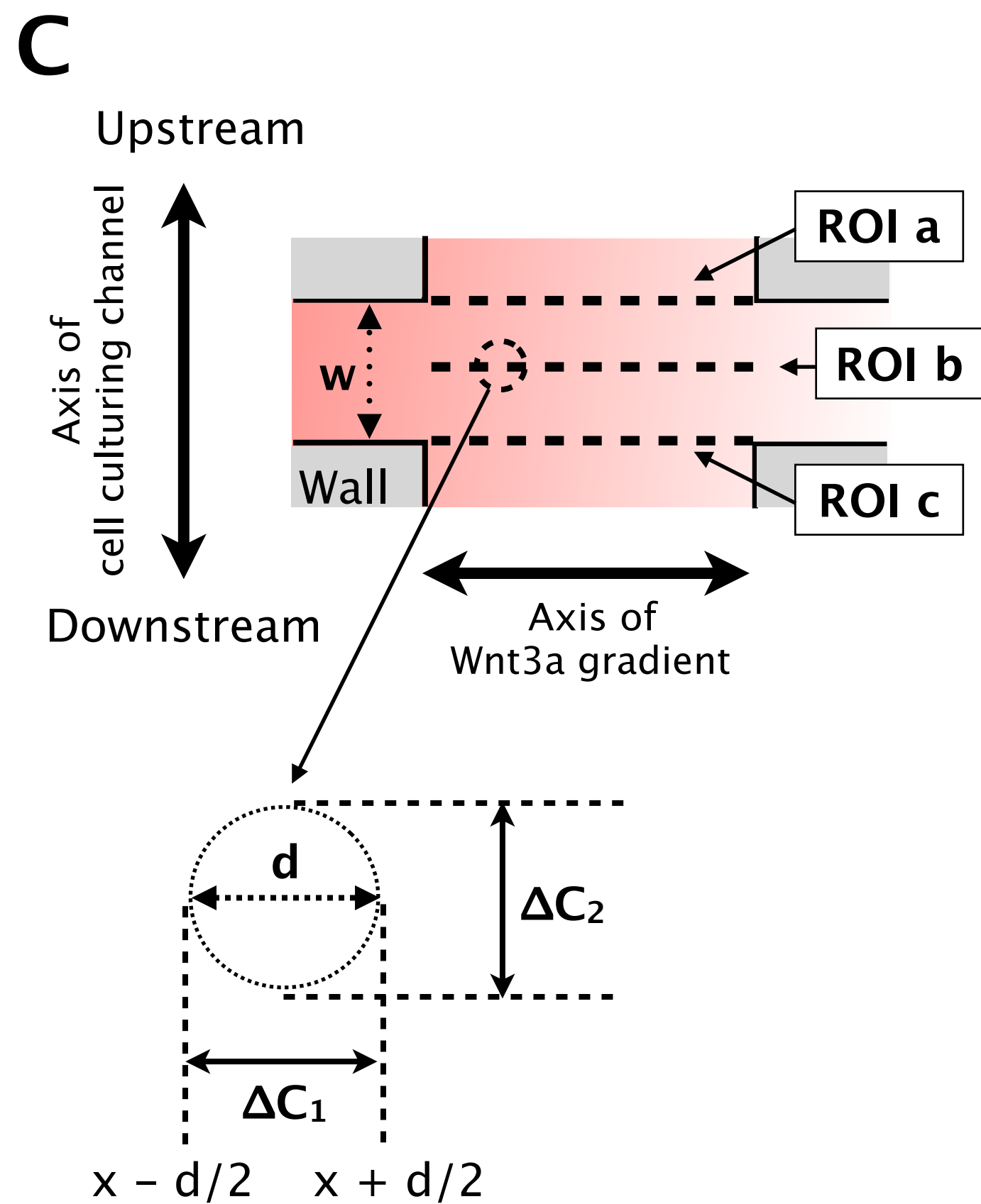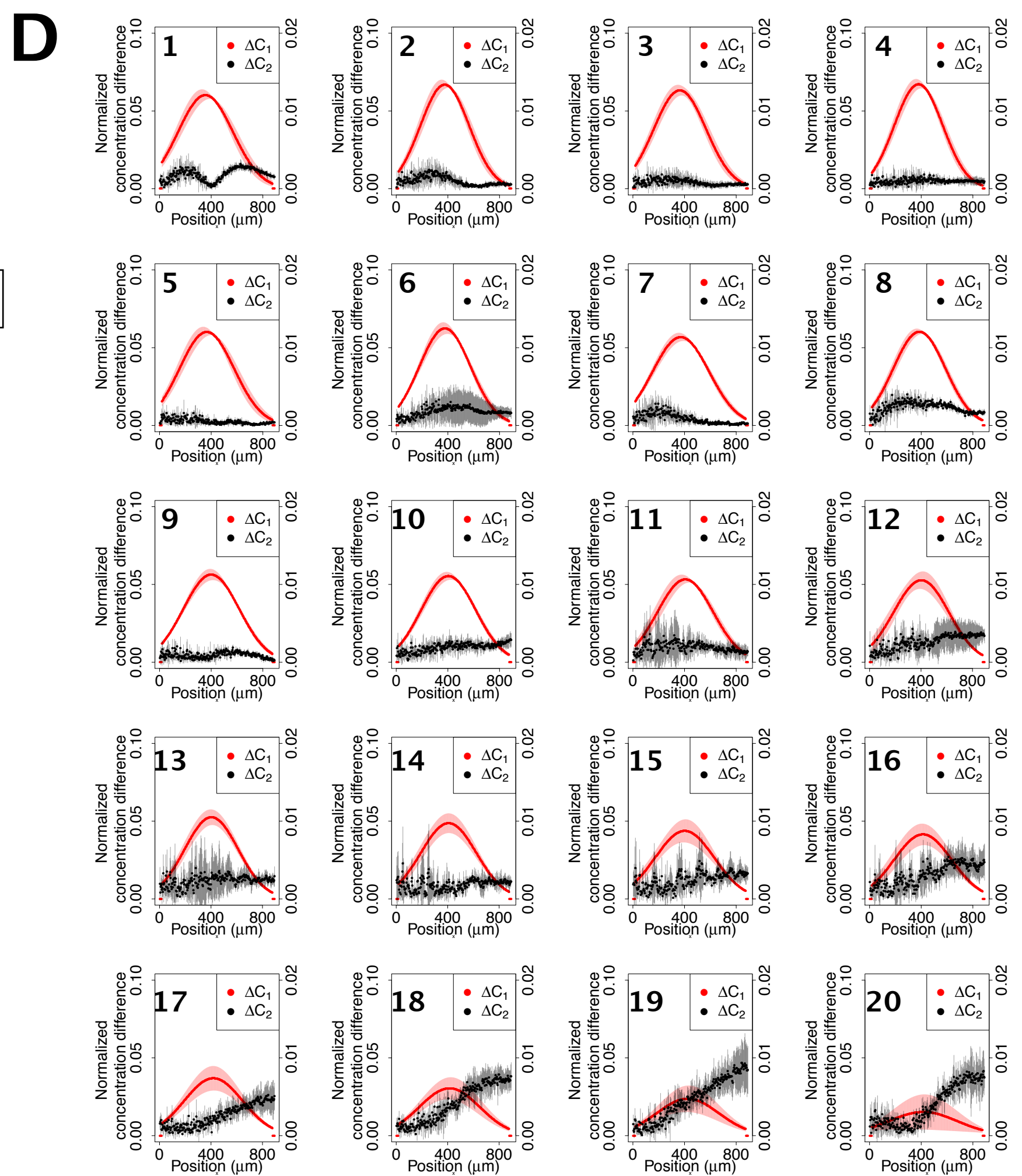

Supplement: Supplementary file 5 — Fig. S5. Profiling of the concentration gradient. (A)Experimental data of normalised intensity (dots) and fitted curve for the data (dashed line) in channel no. 1, at a flow rate of 2 μL·min−1. The residual sum of squares (RSS) value was 0.1059. Error bars show the standard deviation of the normalised intensity. (B) The RSS value in each channel. (C) Diagram of the concentration difference between the two ends of a mitotic cell in relation to the axis of the Wnt3a gradient and the axis of the cell culturing channel (∆C 1 and ∆C 2). ∆C 1 is calculated by CBx−d2−CBx+d22 and ΔC 2 is calculated by CAx−CCx2×d/w. Functions C A,B,C are the concentration gradient equations in (1) for ROI A, B, and C, respectively. Dependent variable x is the position of each channel. Parameter d is the diameter of the mitotic cell (d = 11 pixels, approximately 30 μm). Parameter w is the width of the microgrooves. (D) ∆C 1 (red curve) and ∆C 2 (black curve) in each channel. The horizontal axis indicates the position (μm) across the culturing channel. The vertical axes are the concentration difference of the Wnt3a gradient (left axis: ∆C 1, right axis: ∆C 2). The upper left number shows the channel no. Error bars show the standard deviation of the normalised concentration difference. [file FEB4-8-1920-s005.pdf]

**A**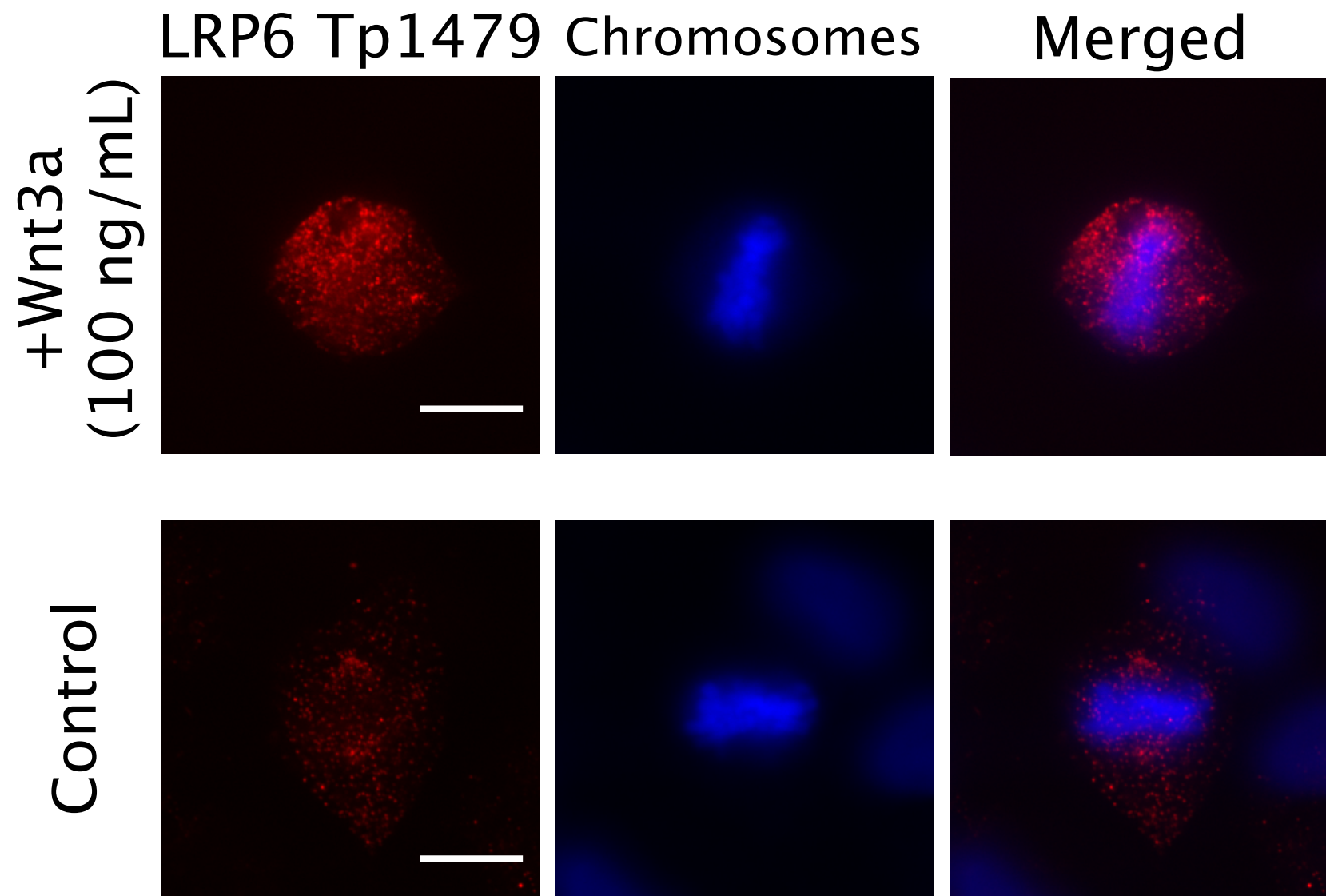**B**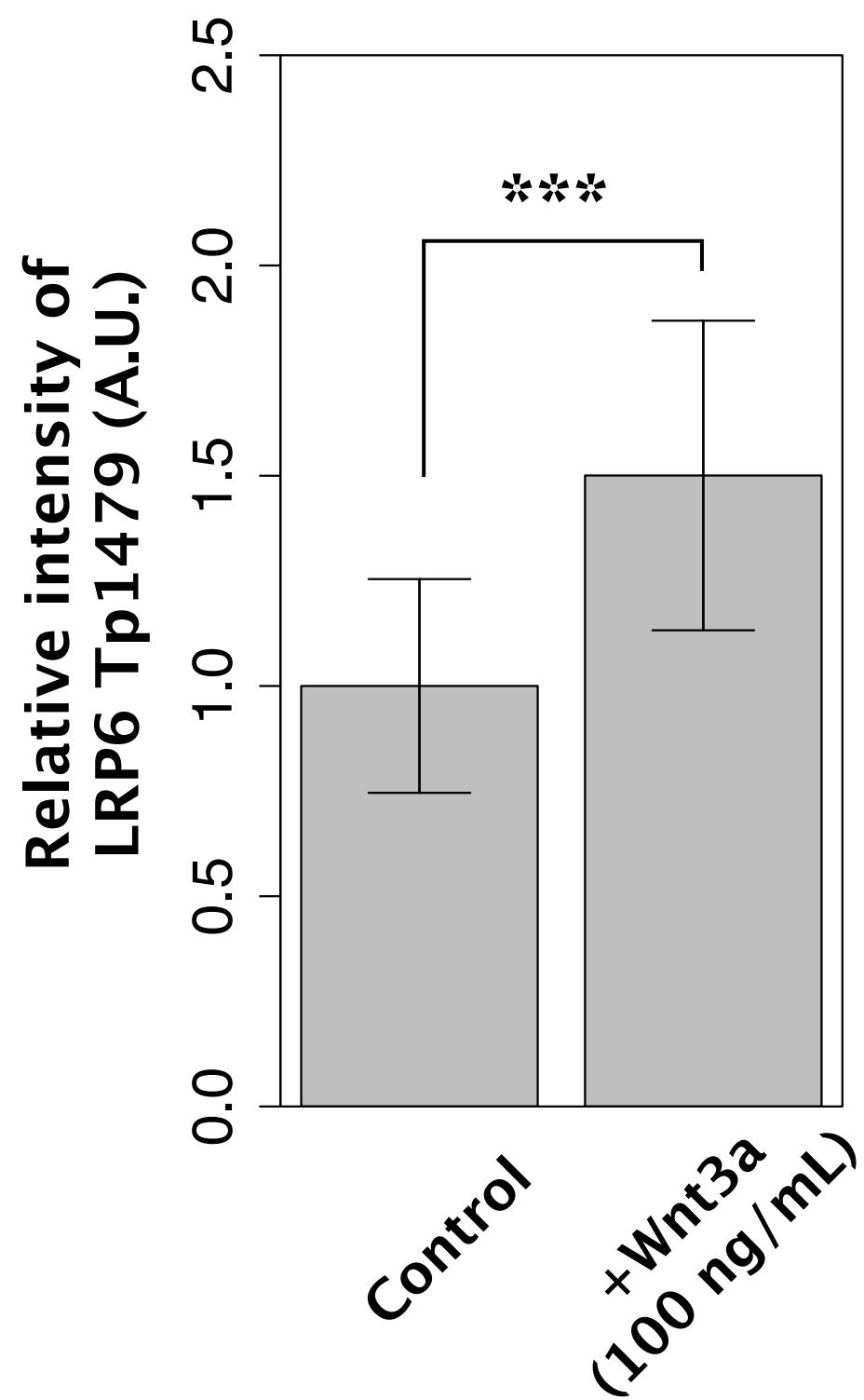

Supplement: Supplementary file 6 — Fig. S6. LRP6 T1479 activation during mitosis by Wnt3a treatment. (A) Immunofluorescence staining of LRP6 Tp1479 during mitosis. Scale bar = 10 μm. (B) Quantification of the relative phosphorylation level of LRP6 Tp1479 (Control; n = 45, Wnt3a; n = 46). Mean ± standard deviation of the mean are displayed. A Student t‐test was performed. ***P < 0.001. [file FEB4-8-1920-s006.pdf]

# A

Upstream

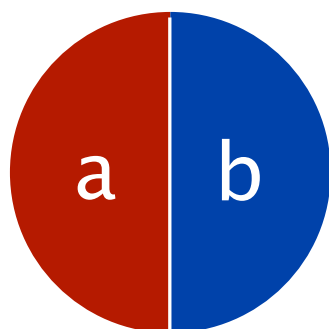

Mitotic cell

Downstream

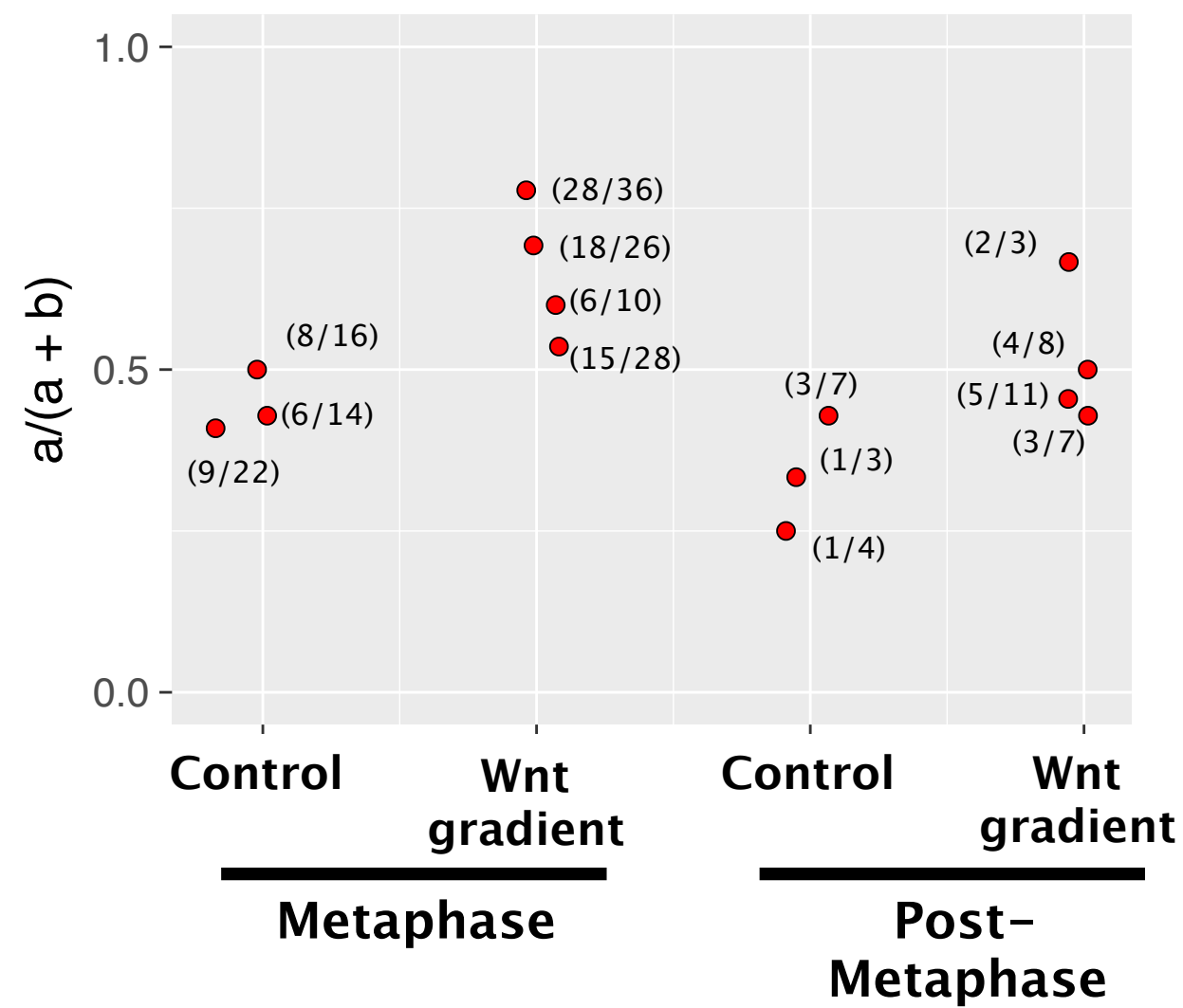

# B

Upstream

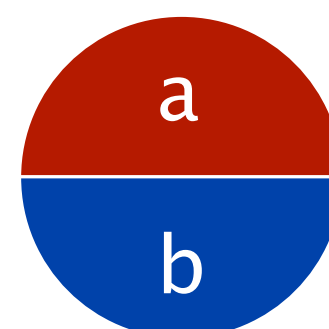

Mitotic cell

Downstream

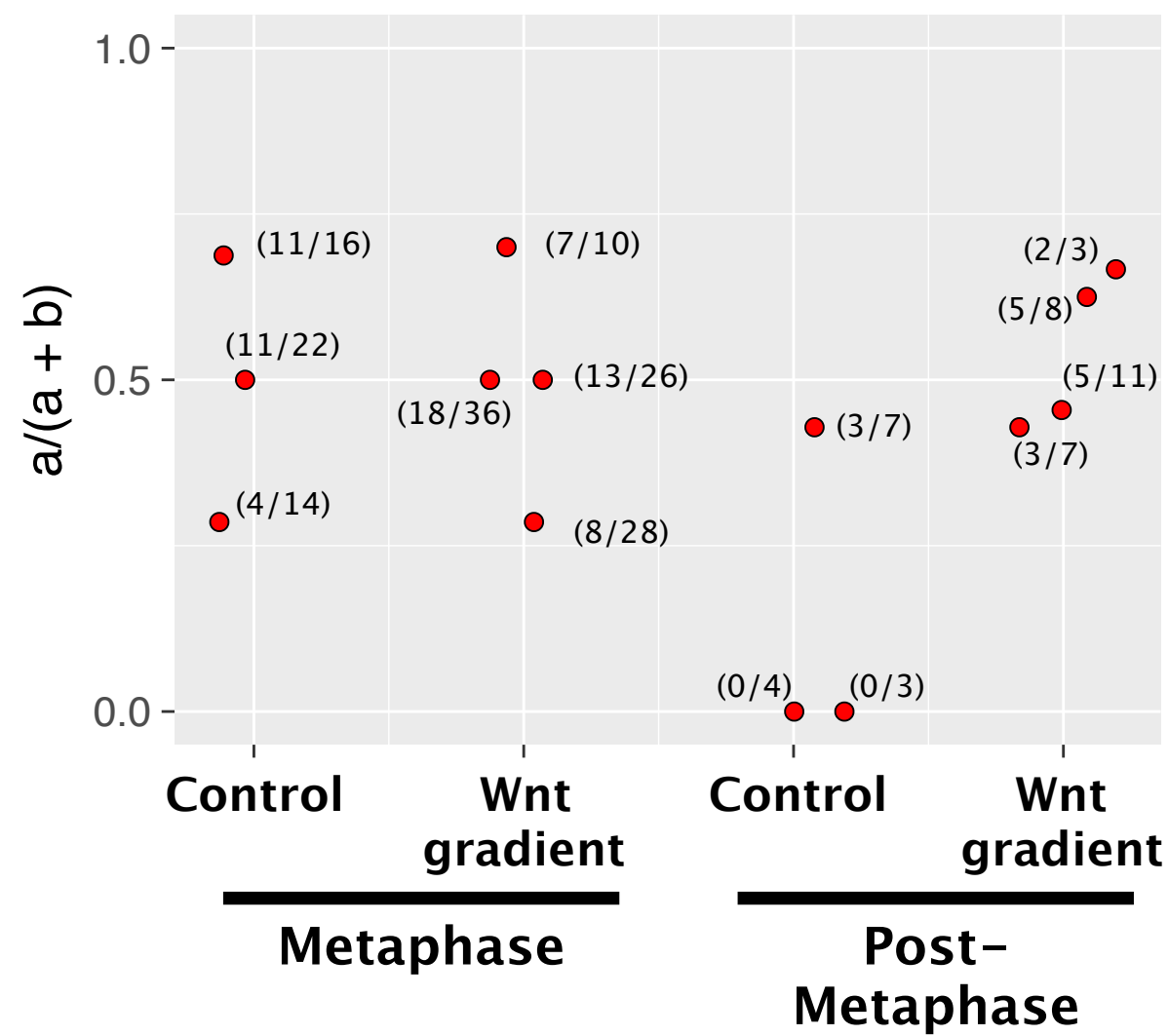

Supplement: Supplementary file 7 — Fig. S7. All plots of each experimental set for Fig. 4E,F. (A) LRP6 Tp1479 at metaphase was localised to the higher concentration side of Wnt3a. The probability (a/(a + b), y‐axis) is the value obtained by dividing the number of cells in which LRP6 Tp1479 was localised at the higher concentration side by the total number of cells in each experiment. (B) The probability (a/(a + b), y‐axis) is the value obtained by dividing the number of cells in which LRP6 Tp1479 was localised to the side of upstream by the total number of cells in each experiment. [file FEB4-8-1920-s007.pdf]

# A

Upstream

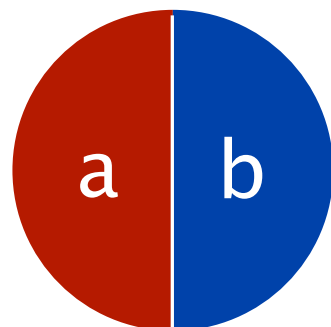

Mitotic cell

Downstream

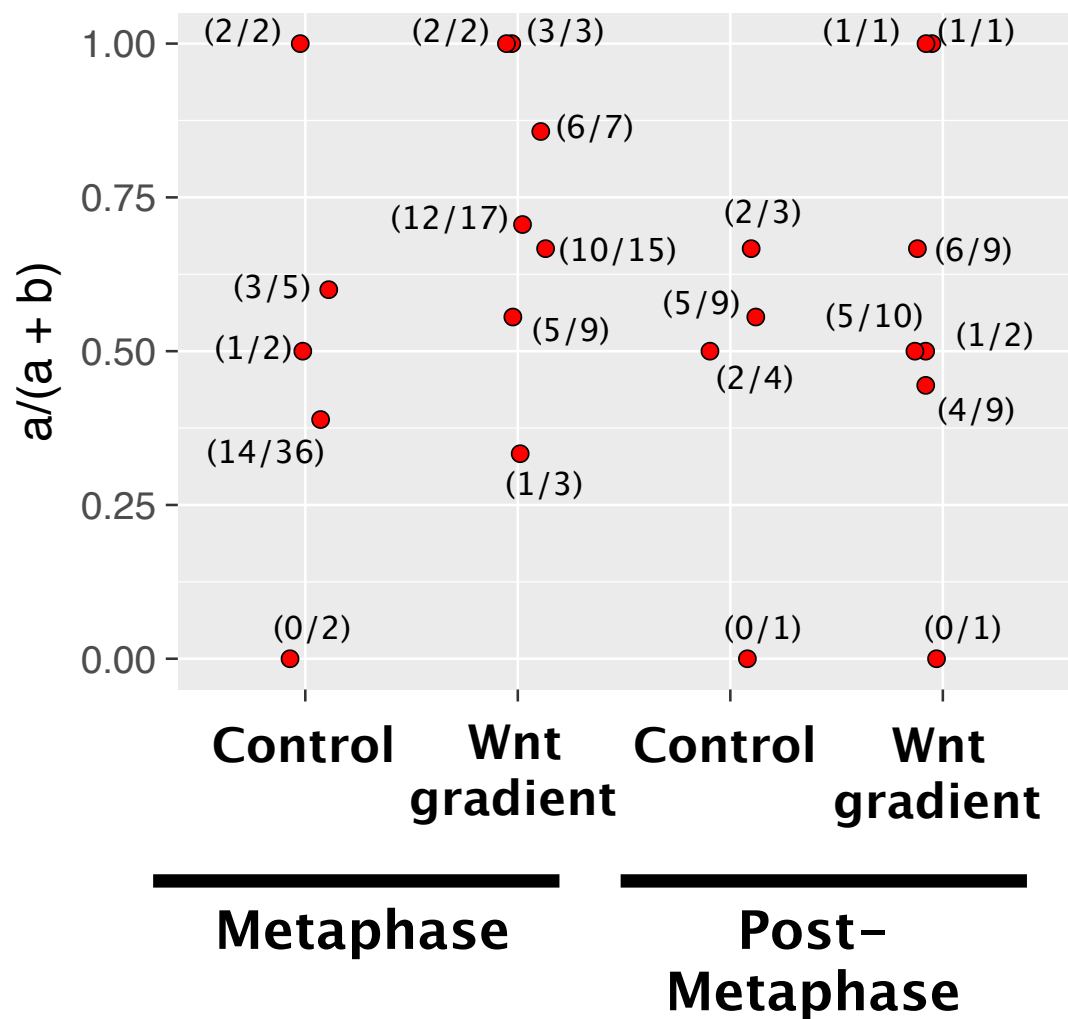

# B

Upstream

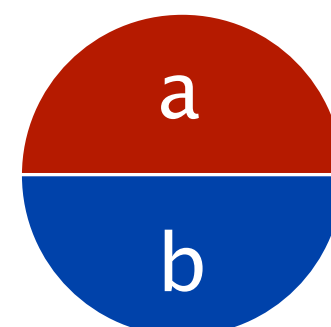

Mitotic cell

Downstream

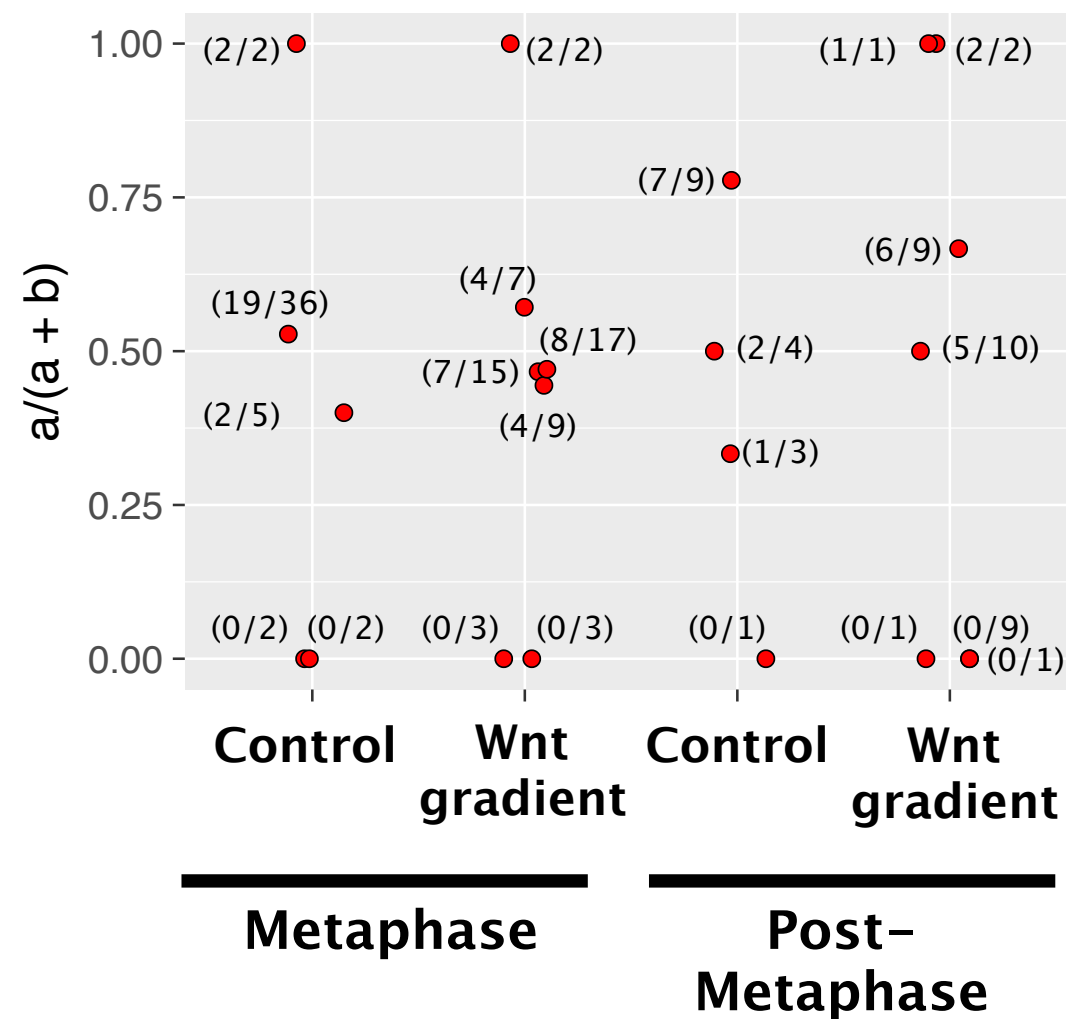

Supplement: Supplementary file 8 — Fig. S8. All plots of each experimental set for Fig. 5D,E. (A) The probability that the orientation of the pole‐to‐pole axis is biased towards the higher concentration side of Wnt3a in each experiment. The value of the axis towards and against 0 degree was counted. Among 6 sets of experiments with metaphase cells in the Wnt gradient, once were less biased towards higher concentration side of Wnt3a (1/3), the other 5 times were biased towards higher concentration side of Wnt3a. (B) The probability of the orientation of the pole‐to‐pole axis towards the upstream side in each experiment. The value towards and against ‐π/2 was counted. [file FEB4-8-1920-s008.pdf]

**A**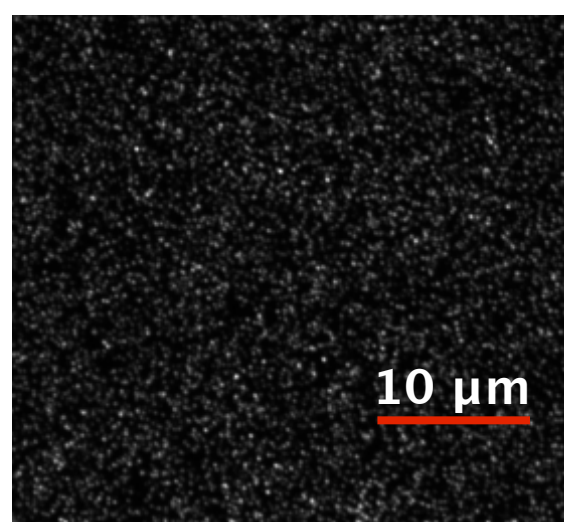

anti-Wnt3a  
on collagen coated glass  
(Control)

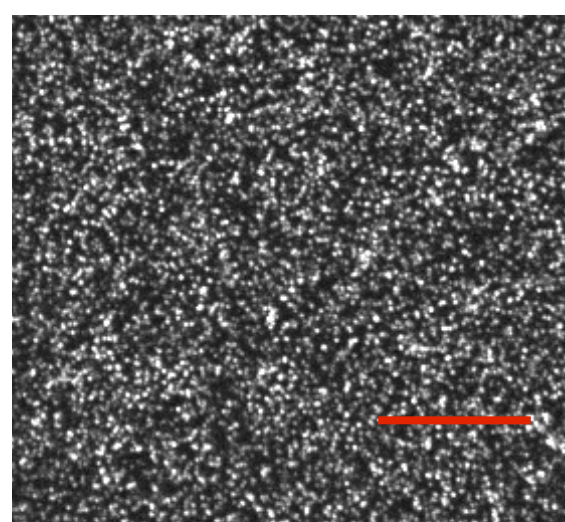

Wnt3a +  
anti-Wnt3a  
on collagen coated glass

**B**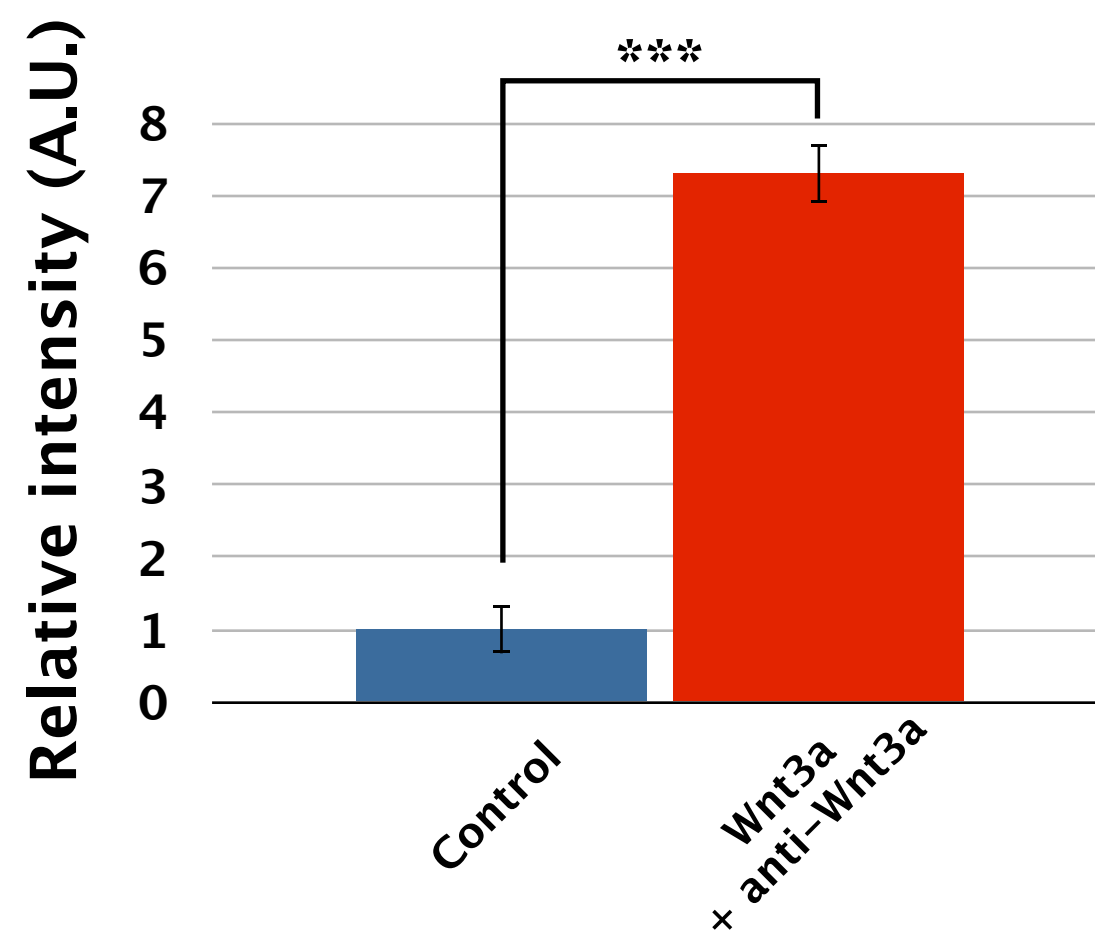**C**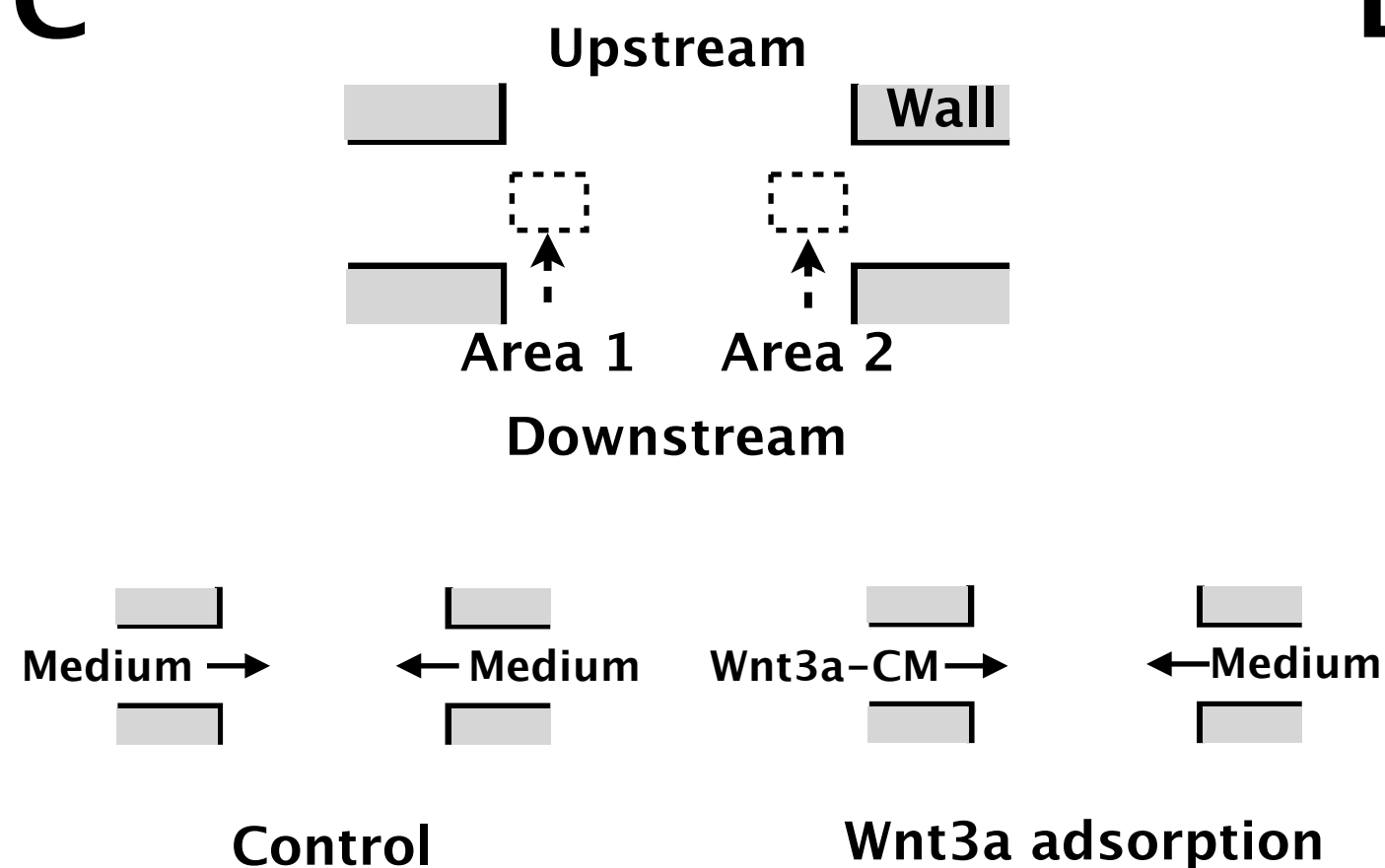**D**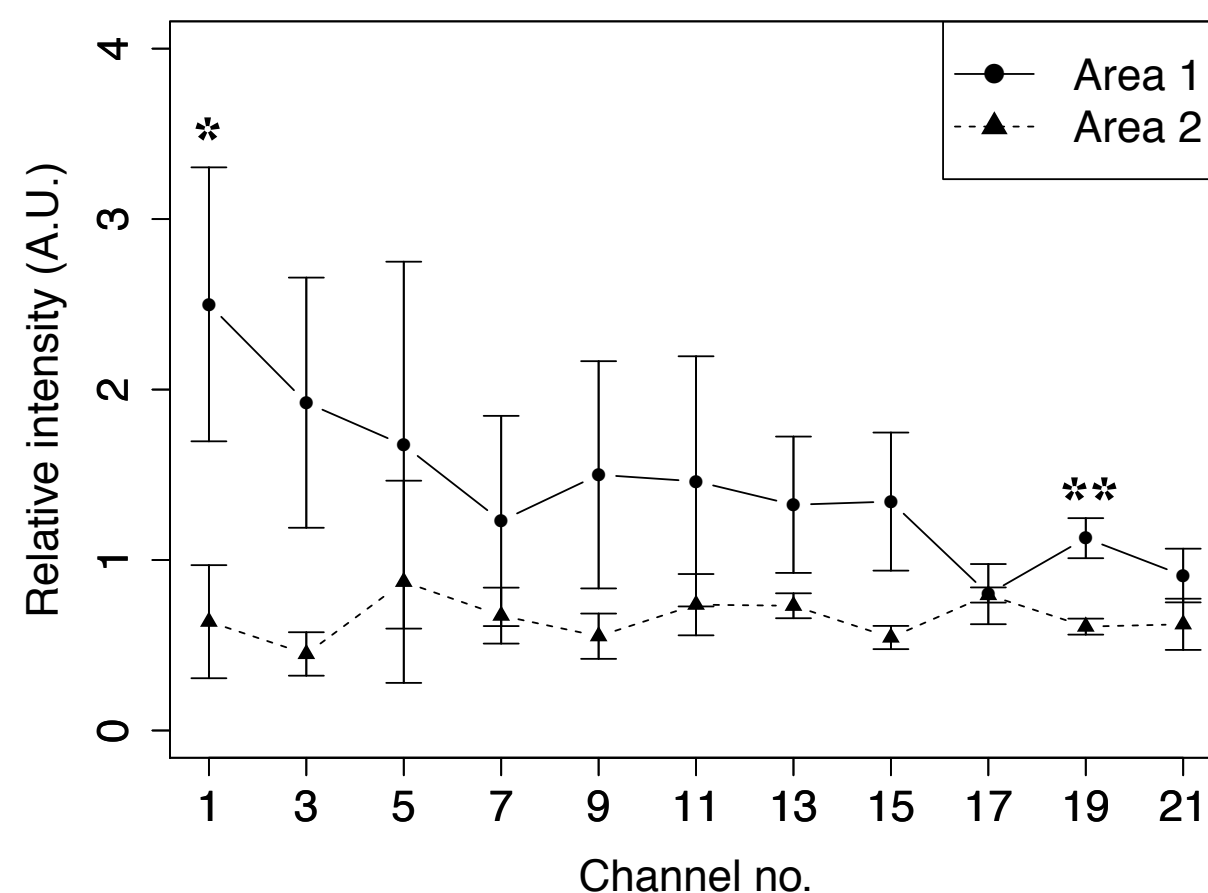**E**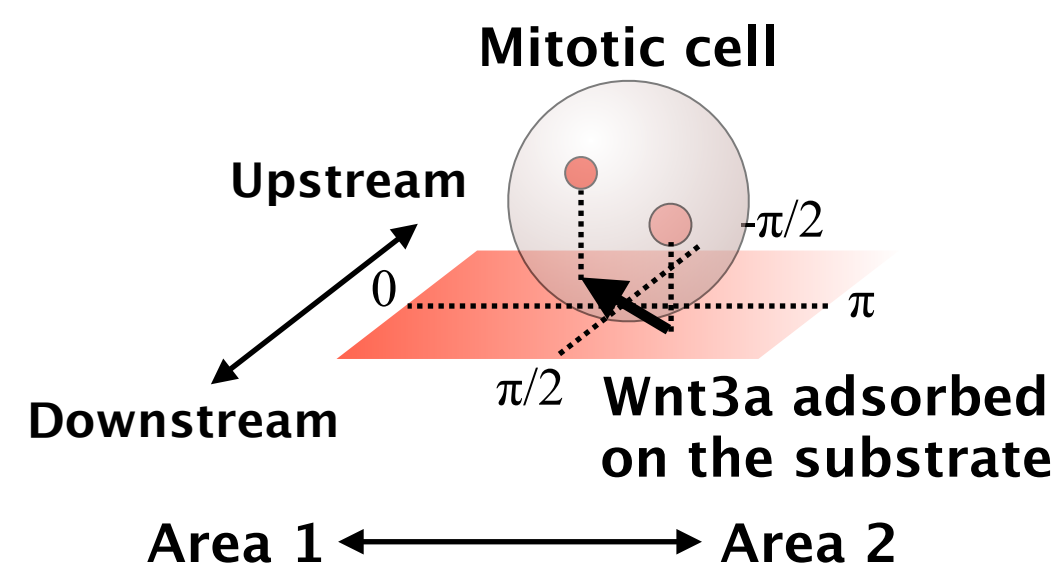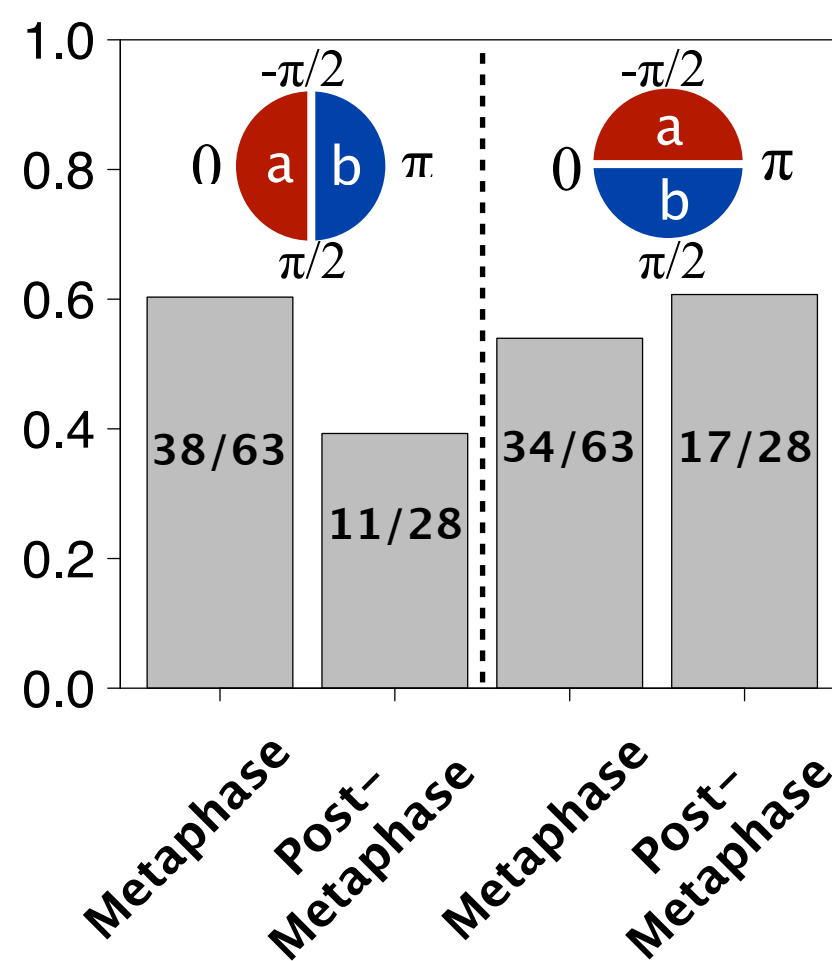**F**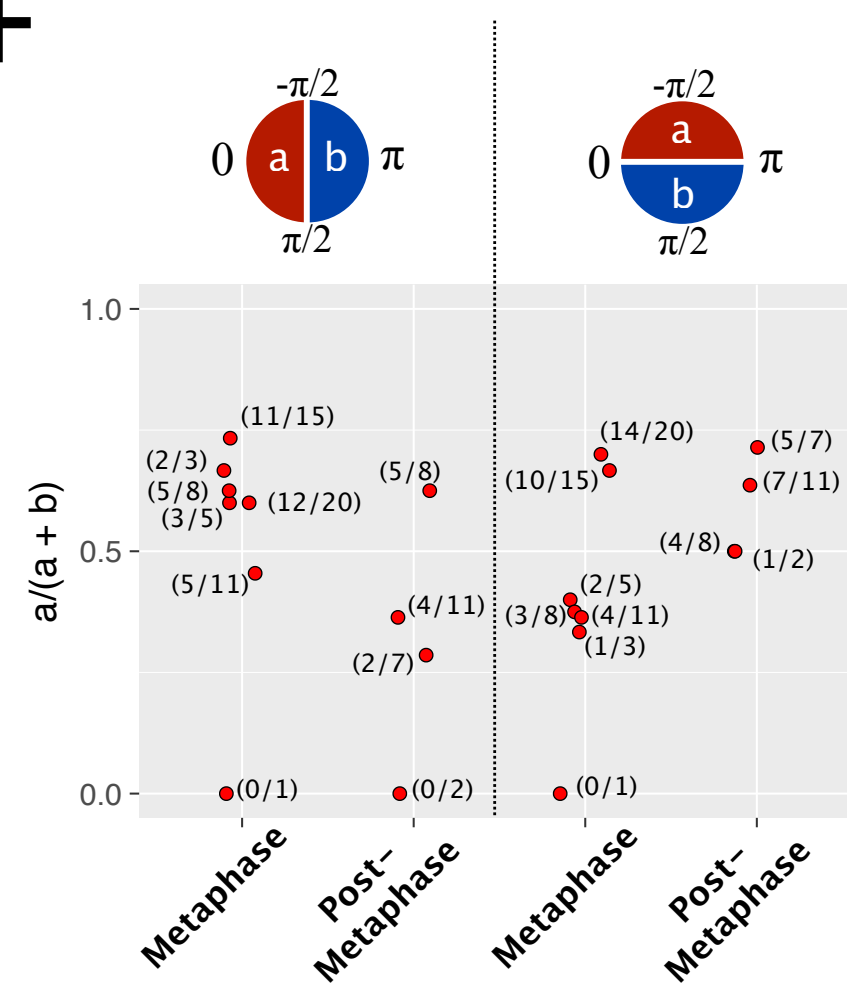

Supplement: Supplementary file 9 — Fig. S9. Wnt3a adsorption on collagen. (A)Fluorescent images of collagen‐coated glass and Wnt3a‐adsorbed collagen‐coated glass. The collagen‐coated glass was incubated with either culturing medium only or Wnt3a‐CM. After incubation, Wnt3a adsorption was detected by anti‐Wnt3a antibodies. (B) Quantification of Wnt3a adsorption. The intensity of each experiment was obtained by calculating the average intensity of five fluorescent images. The relative intensity was obtained by dividing each intensity by the average intensity of the control. The individual experiments for each condition were repeated three times. ***P < 0.001: Student t‐test. (C) Diagram of measuring areas in the culturing channel. The relative intensity was measured in the areas near the microgrooves (Area 1 and Area 2). For the control experiment in the culturing channel, culturing medium was perfused from both sides. For the Wnt3a adsorption experiment in the culturing channel, Wnt3a‐CM and culturing medium was perfused from each side of the culturing channel. (D) Profiling the Wnt3a adsorption experiment. Black dots and triangles indicate the average of the relative intensity in Area 1 and Area 2 after 12 h of perfusion at a flow rate of 2 μL·min−1. Error bars show the standard deviation of the relative intensity. *P < 0.05, **P < 0.01: Student t‐test. (E) The cells were cultured on Wnt3a adsorbed collagen‐coated glass. The probability of the orientation of the pole‐to‐pole axis towards Area 1 and the upstream side of Wnt3a adsorbed collagen‐coated glass is shown in the right panel. There was no significant bias in the orientation of the pole‐to‐pole axis using a two‐sided, exact binomial test. (F) All plots of each experimental set for (E). [file FEB4-8-1920-s009.pdf]

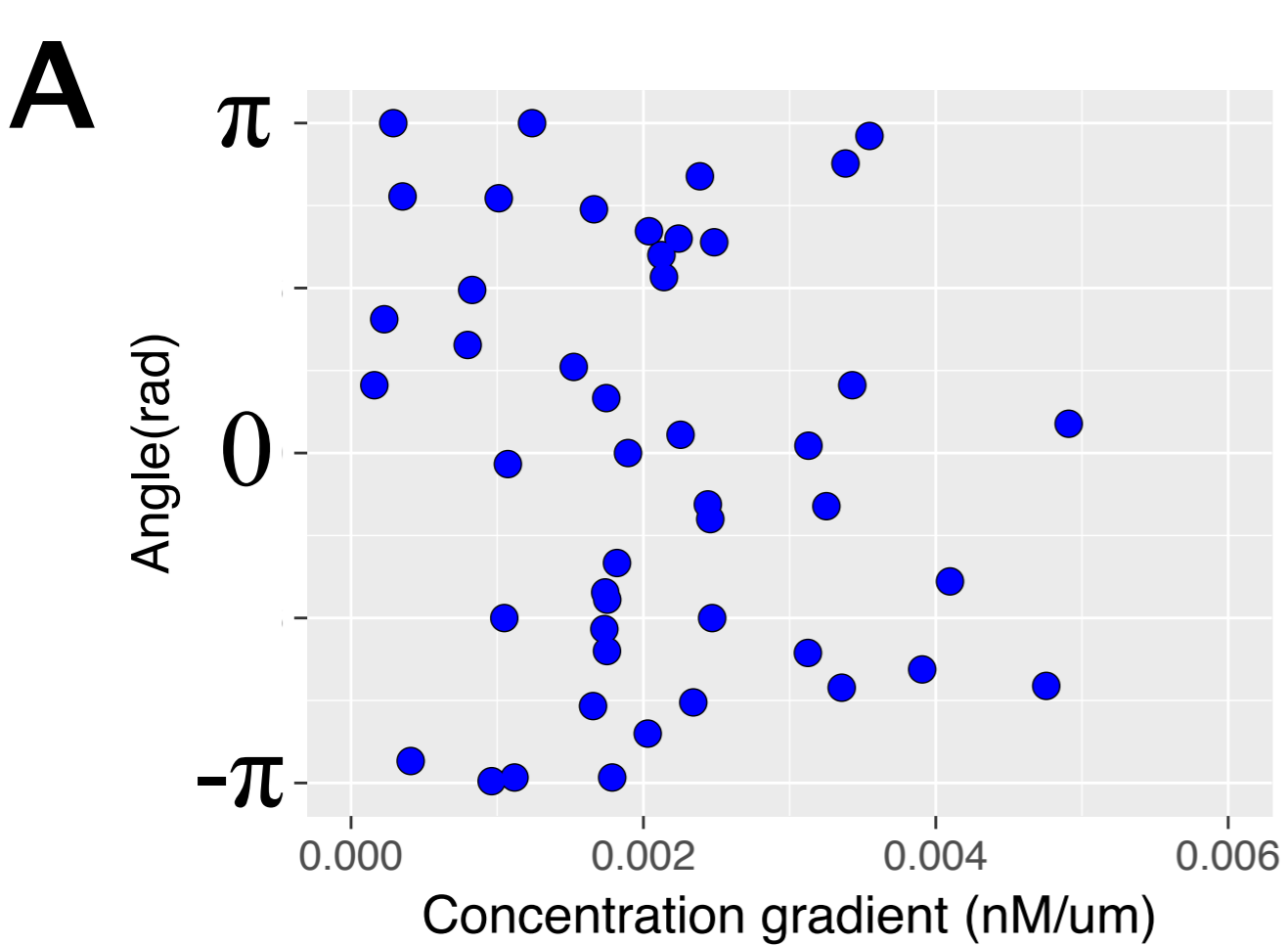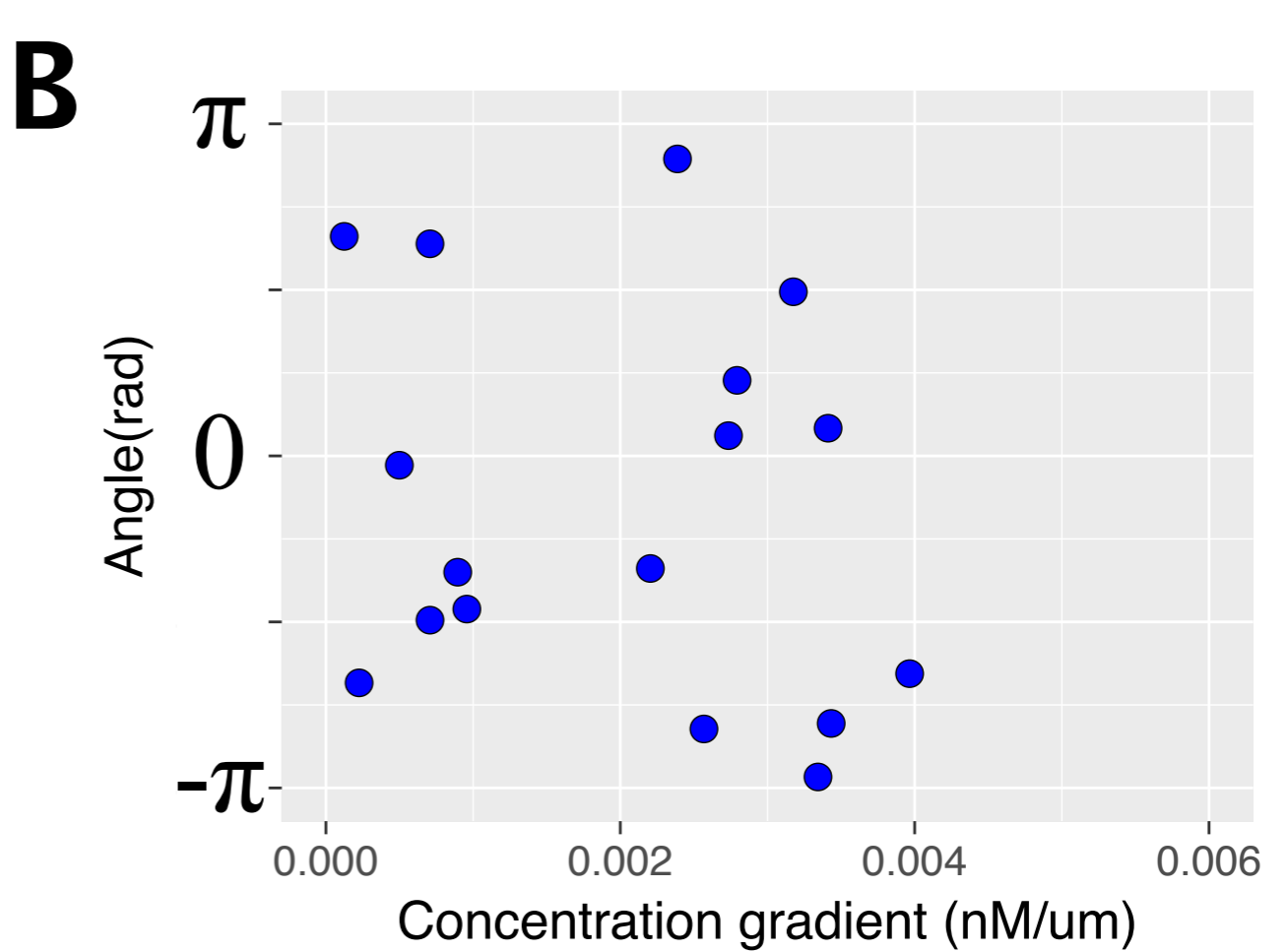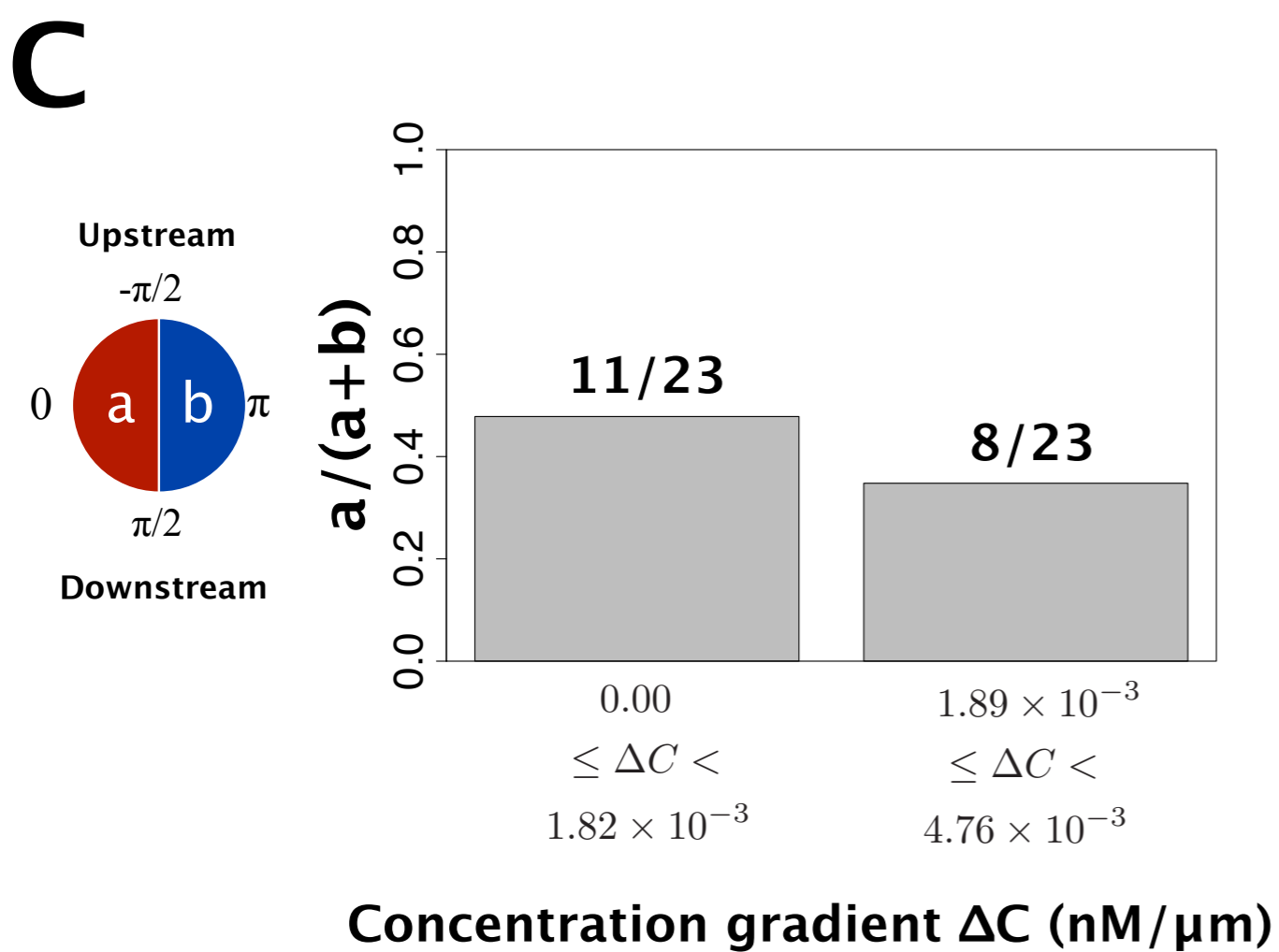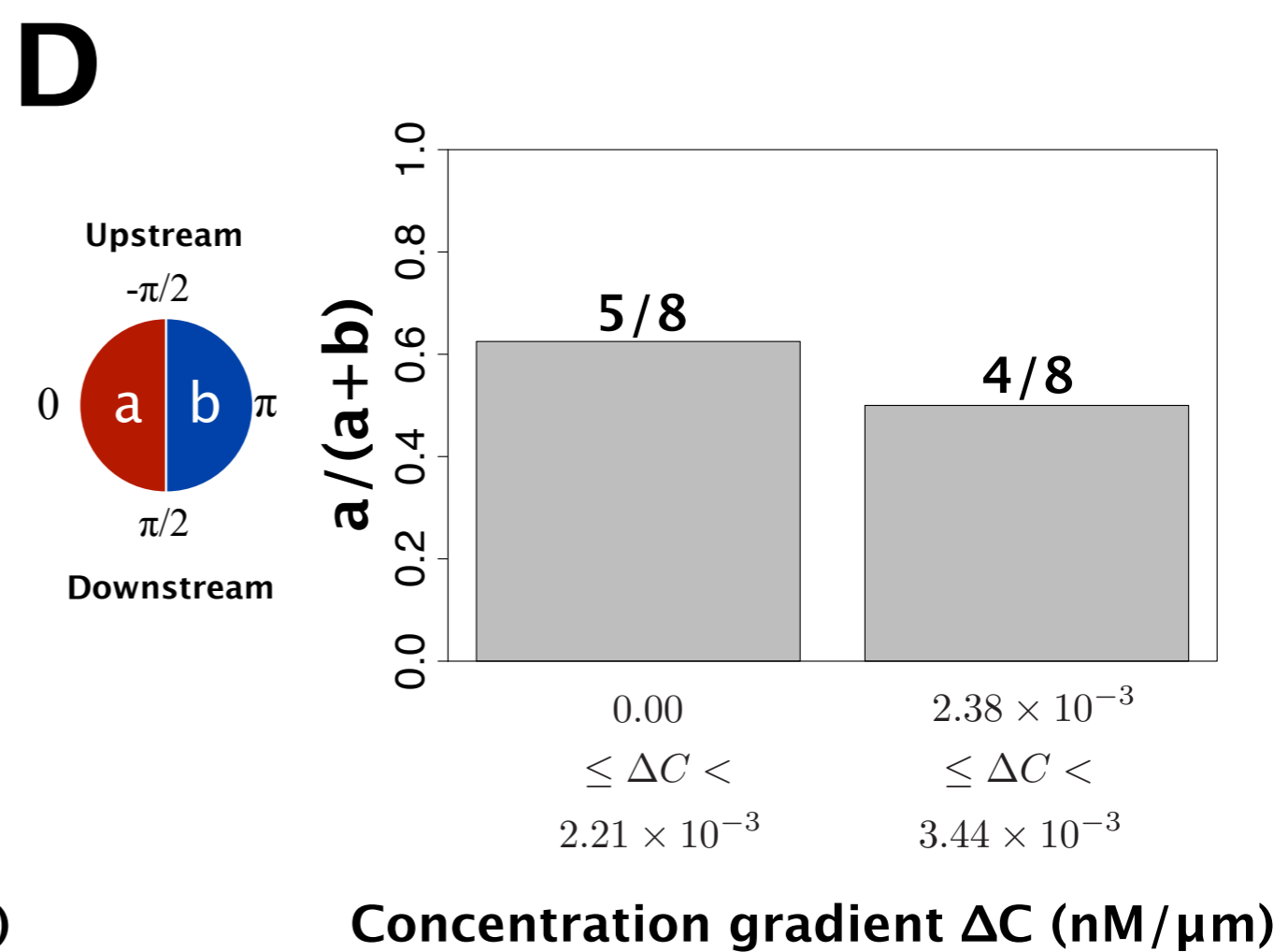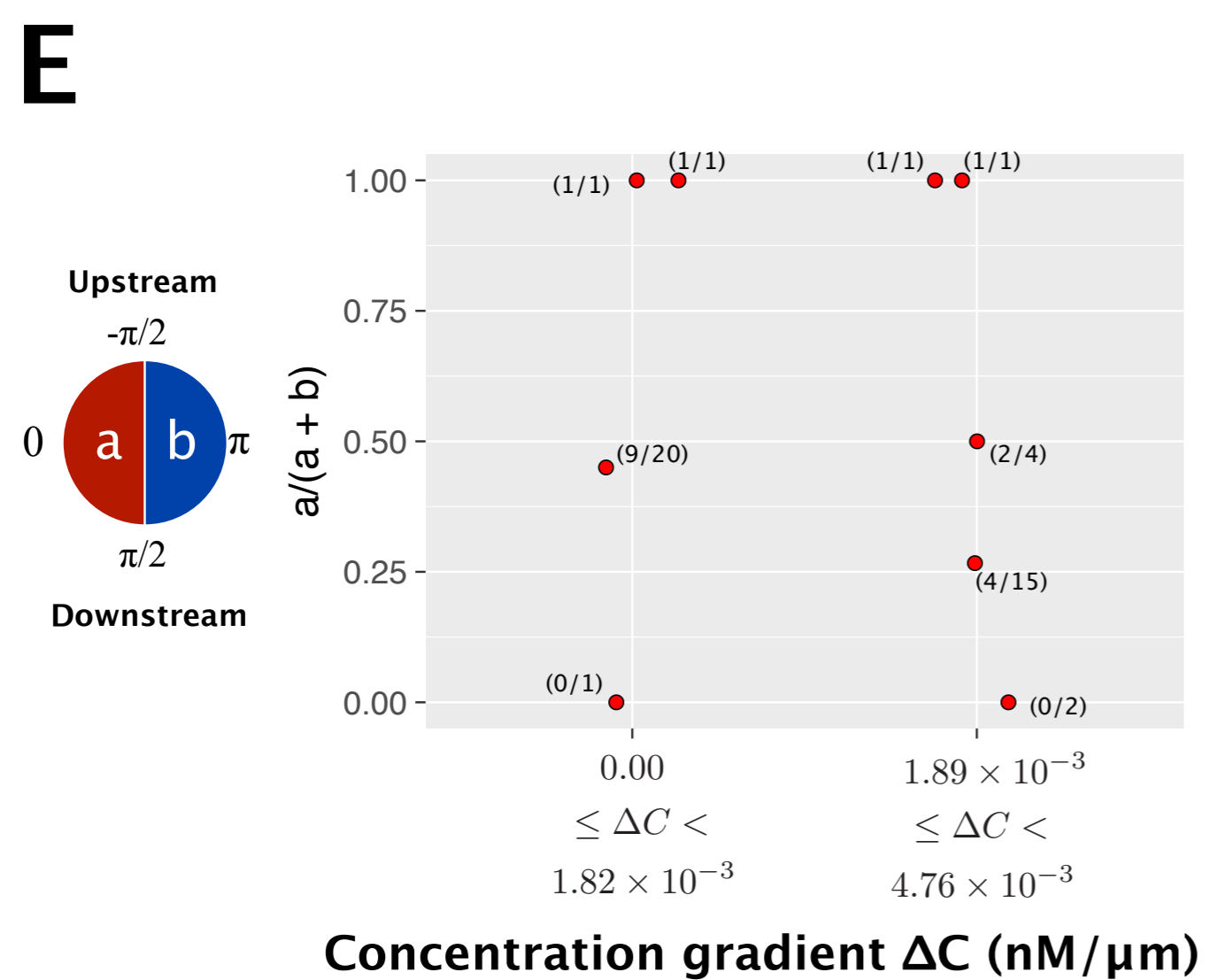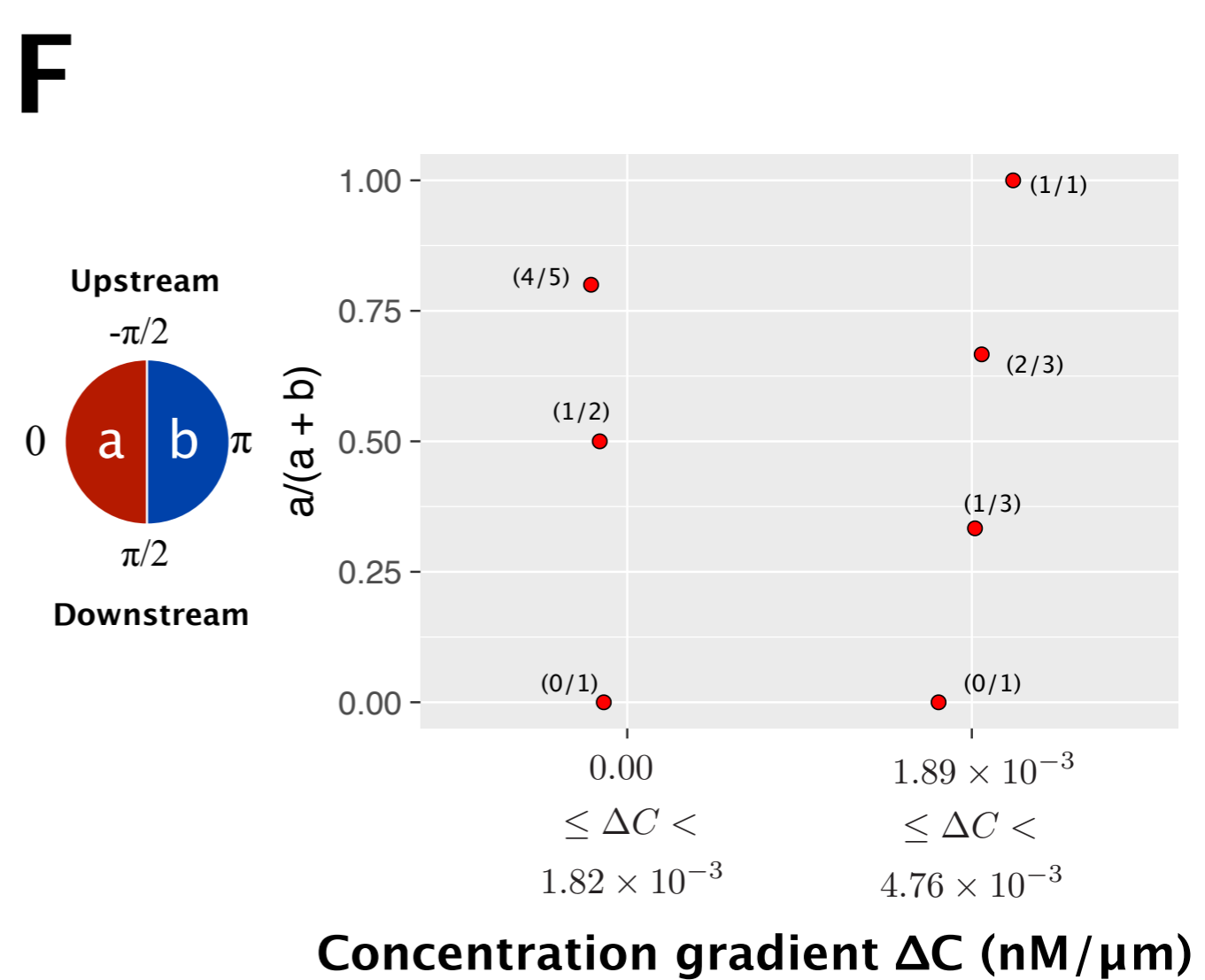

Supplement: Supplementary file 11 — Fig. S11. Relationship between the angle of the pole‐to‐pole axis and the culturing environment without a Wnt3a concentration gradient. A plot of the angle of the pole‐to‐pole axis without a Wnt3a gradient (∆C) during (A) metaphase and (B) postmetaphase in Fig. 5D. We calculated a virtual concentration gradient when Wnt3a‐CM was injected. (C) Probability that the pole‐to‐pole axis orientates towards the microgrooves which drain Wnt3a‐CM during metaphase, and (D) postmetaphase. We performed a two‐sided, exact binomial test and there was no significant difference. (E and F) All plots of each experimental set for (C) and (D). [file FEB4-8-1920-s011.pdf]

**A****Upstream**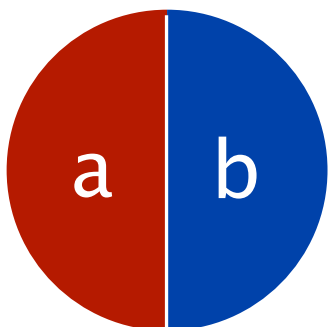**Mitotic cell****Downstream**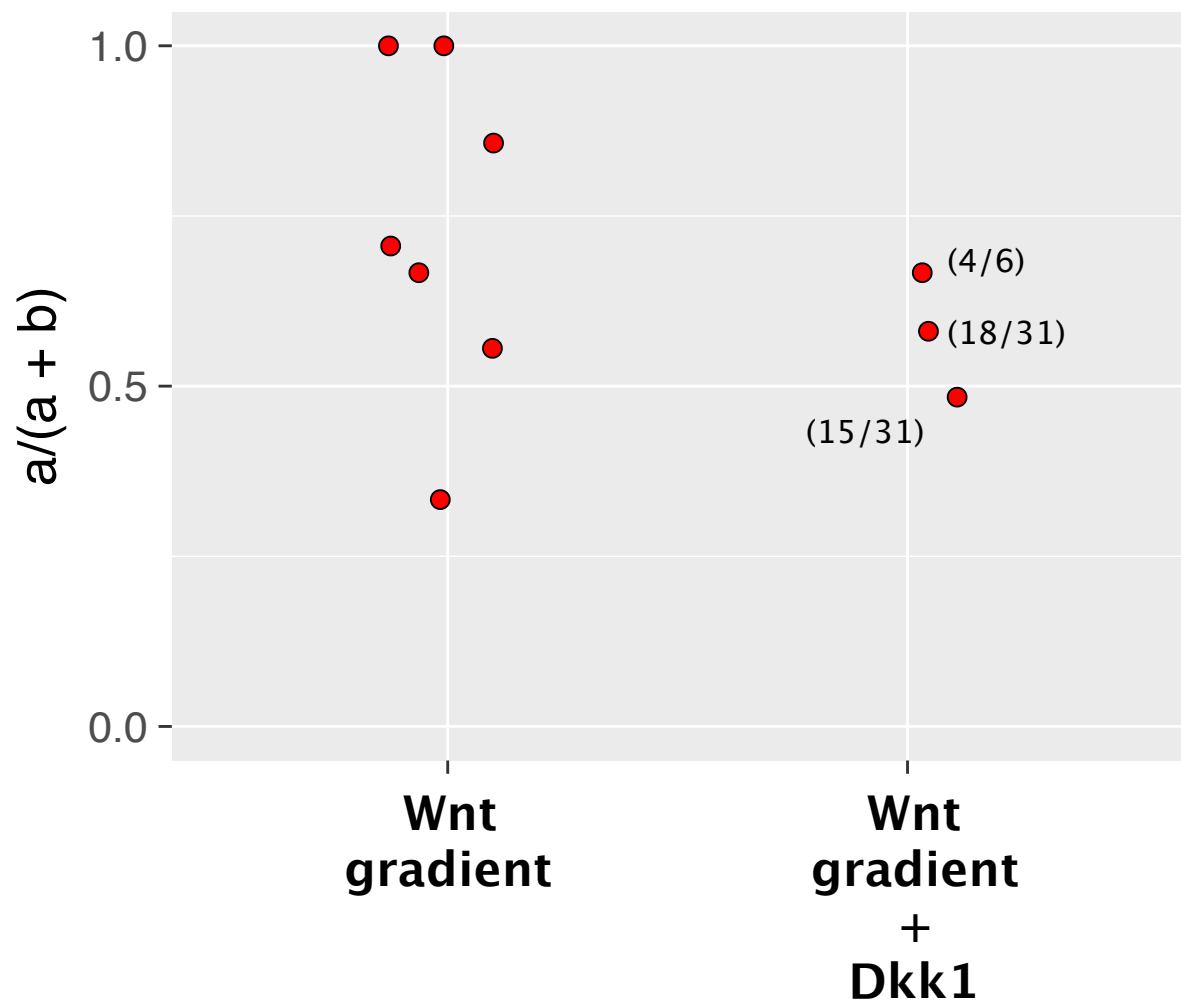**B****Upstream**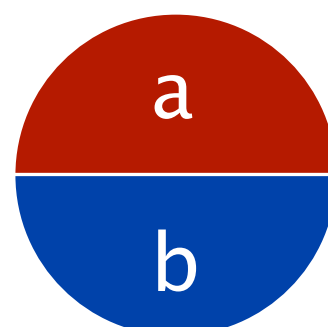**Mitotic cell****Downstream**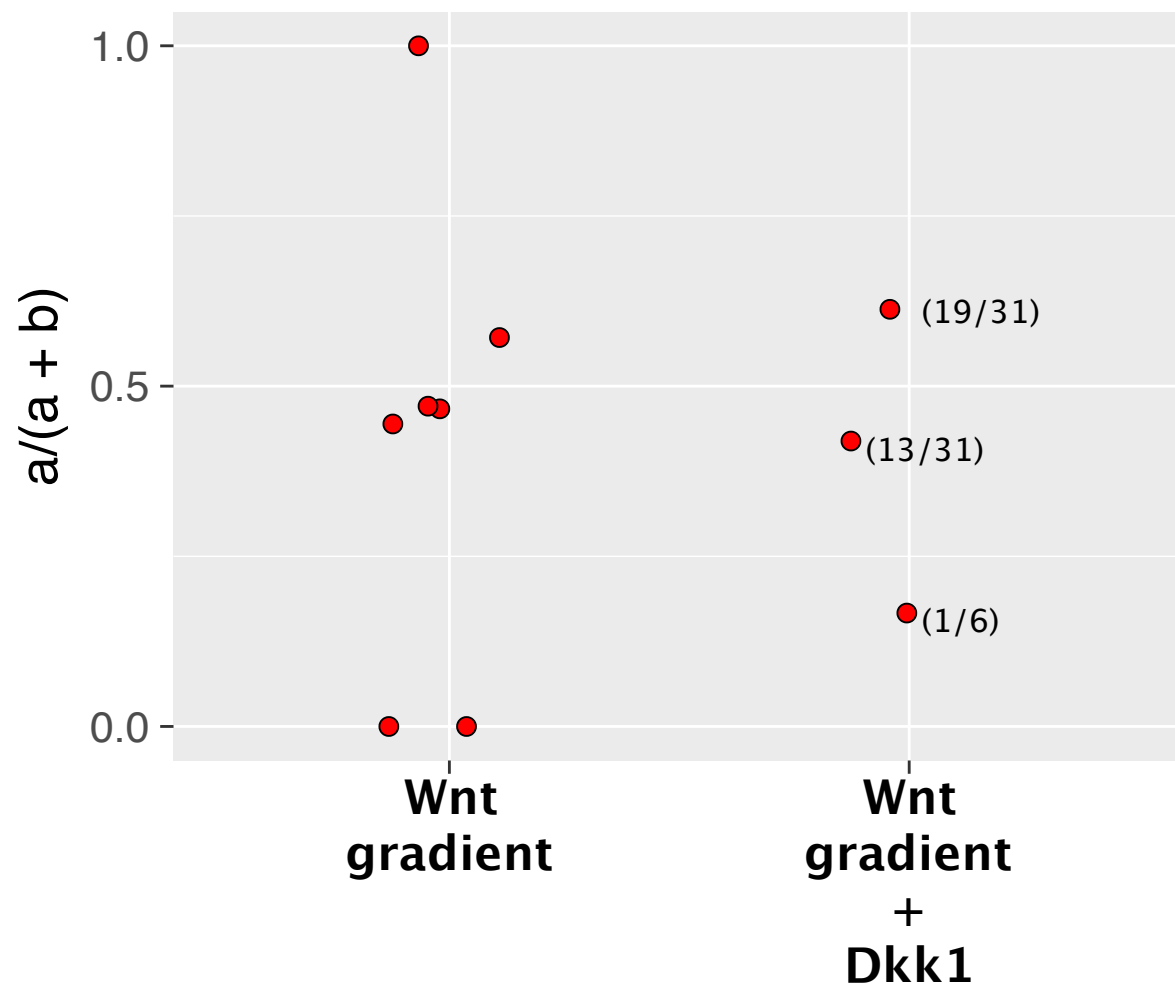

Supplement: Supplementary file 12 — Fig. S12. All plots of each experimental set for Fig. 6F,G. (A) The biased ratio of the pole‐to‐pole axis to the high Wnt3a concentration side with the addition of Dkk1 in metaphase. (B) The biased ratio of the pole‐to‐pole axis to medium flow direction when Dkk1 was added during metaphase. [file FEB4-8-1920-s012.pdf]
