# Supplementary material for: Quantitative analysis of sensitivity to a Wnt3a gradient in determination of the pole‐to‐pole axis of mitotic cells by using a microfluidic device
Source: FEBS Open Bio. 2018 Nov 9;8(12):1920–35. doi: 10.1002/2211-5463.12525 (PMC6275273; doi:10.1002/2211-5463.12525)
Supplement: Supplementary file 4 — Fig. S4. Blocking nonspecific protein adsorption on the PDMS surface. (A) Microscopic images of adsorbed BSA‐FITC on the straight microchannel. Nonspecific adsorption of BSA‐FITC on the untreated surface (left) and the MPC polymer dissolved in absolute ethanol‐coated surface (middle). The surface coated with MPC polymer dissolved in a mixture of chloroform and ethanol (right) reduces BSA‐FITC adsorption. Scale bar = 200 μm. (B) Quantification of the adsorption of BSA protein. (C, D) The device, whose channel was coated with MPC polymer dissolved in a mixture of chloroform and ethanol, was fixed with methanol (C), or with PFA (D), and washed with ethanol followed by heat sterilisation. Blocking capacity was retained through several repeated wash and heat cycles. The y‐axes of graphs in C and D refer to the relative intensity of BSA‐FITC fluorescence after the indicated number of wash and heat cycles to the intensity after the first cycle. [file FEB4-8-1920-s004.pdf]

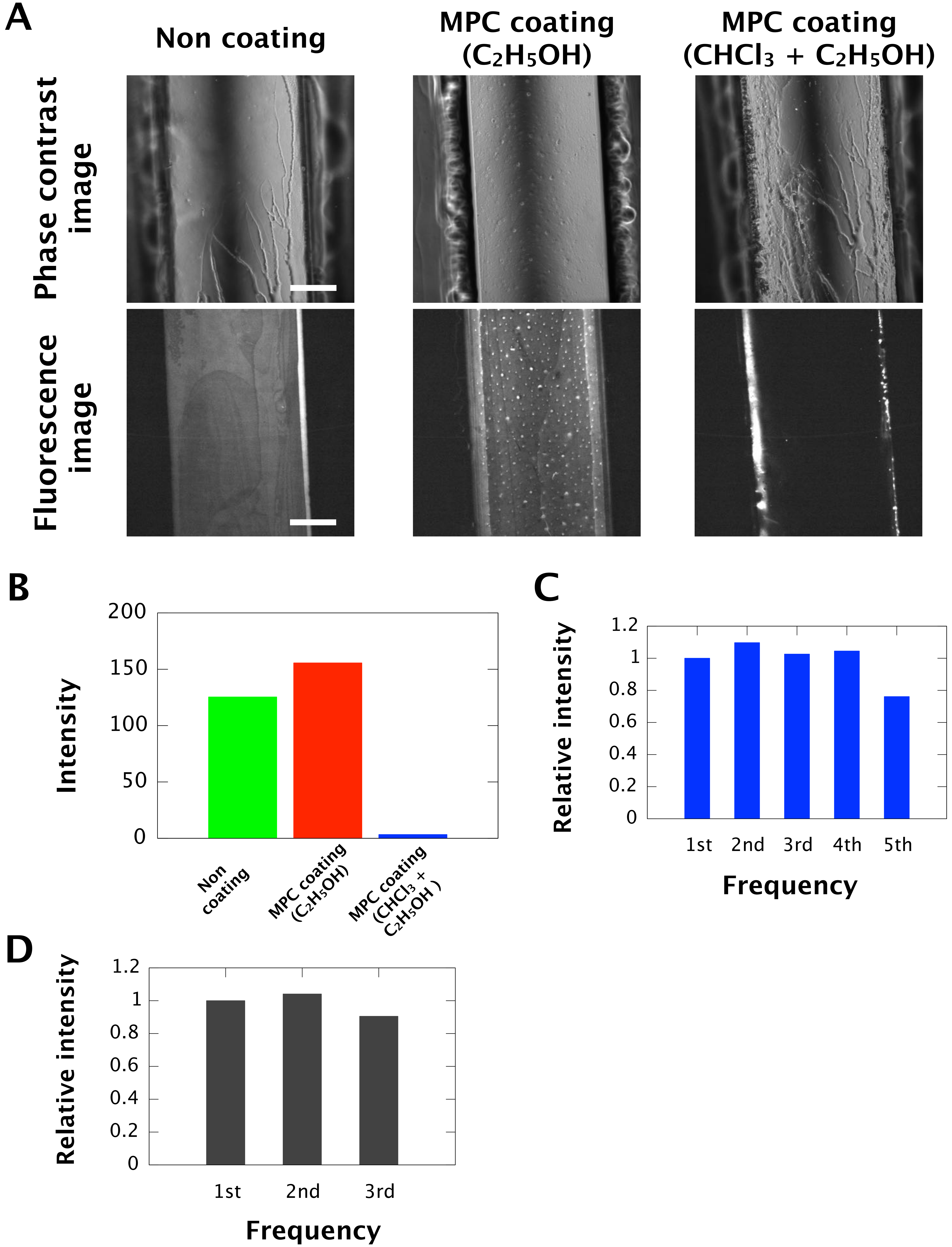

A
Non coating
MPC coating (C_2H_5OH)
MPC coating ($CHCl_3$ + C_2H_5OH)
Phase contrast image
Fluorescence image
B
Intensity
200
150
100
50
0
Non coating
MPC coating (C_2H_5OH)
MPC coating ($CHCl_3$ + C_2H_5OH)
C
Relative intensity
1.2
1
0.8
0.6
0.4
0.2
0
1st
2nd
3rd
4th
5th
Frequency
D
Relative intensity
1.2
1
0.8
0.6
0.4
0.2
0
1st
2nd
3rd
Frequency
