# Supplementary material for: Quantitative analysis of sensitivity to a Wnt3a gradient in determination of the pole‐to‐pole axis of mitotic cells by using a microfluidic device
Source: FEBS Open Bio. 2018 Nov 9;8(12):1920–35. doi: 10.1002/2211-5463.12525 (PMC6275273; doi:10.1002/2211-5463.12525)
Supplement: Supplementary file 10 — Fig. S10. All plots of each experimental set for Fig. 6C,D (A) The biased ratio of the pole‐to‐pole axis in metaphase cells exposed to the high Wnt3a‐concentration side in each experiment. The number of concentration gradient ranges was divided into four. (B) The biased ratio of the pole‐to‐pole axis in postmetaphase cells exposed to the high Wnt3a‐concentration side in each experiment. [file FEB4-8-1920-s010.pdf]

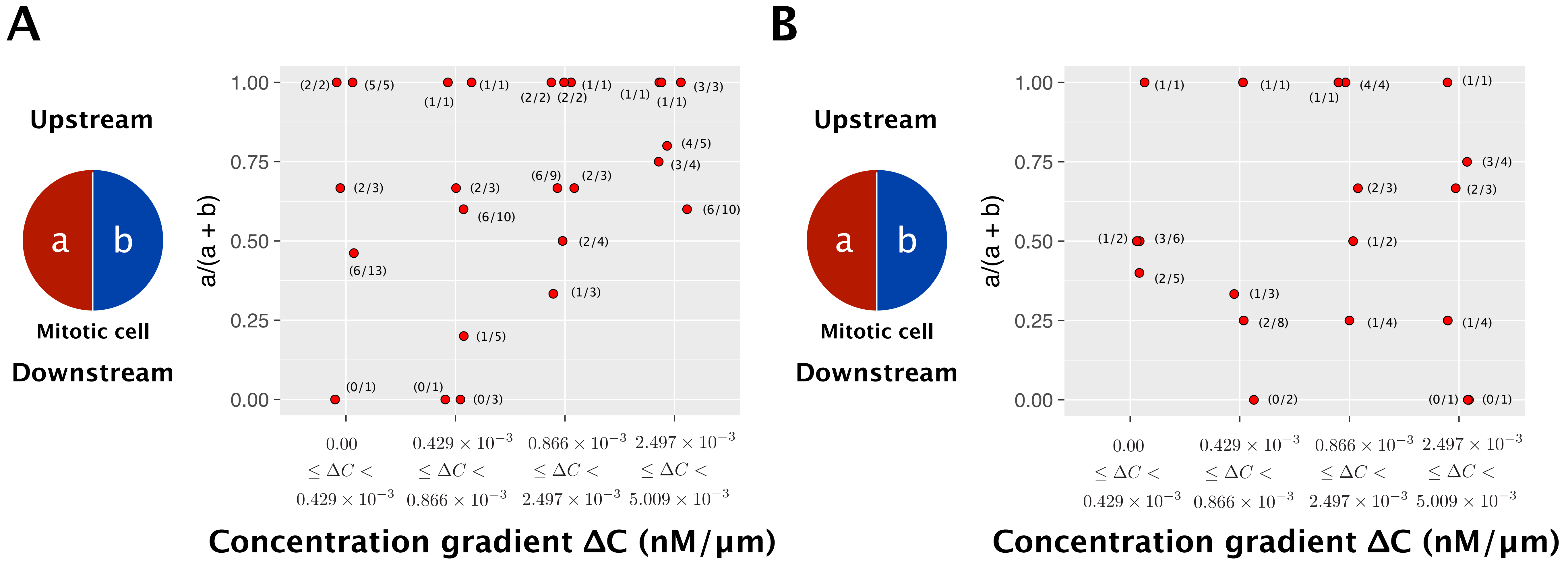
A
Upstream
a
b
Mitotic cell
Downstream
a/(a + b)
1.00
0.75
0.50
0.25
0.00
(2/2)
(5/5)
(1/1)
(1/1)
(2/2)
(2/2)
(1/1)
(1/1)
(1/1)
(3/3)
(4/5)
(3/4)
(2/3)
(2/3)
(6/9)
(2/3)
(6/10)
(6/10)
(2/4)
(6/13)
(1/3)
(1/5)
(0/1)
(0/1)
(0/3)
$0.00 \leq \Delta C < 0.429 \times 10^{-3}$
$0.429 \times 10^{-3} \leq \Delta C < 0.866 \times 10^{-3}$
$0.866 \times 10^{-3} \leq \Delta C < 2.497 \times 10^{-3}$
$2.497 \times 10^{-3} \leq \Delta C < 5.009 \times 10^{-3}$
Concentration gradient ΔC (nM/μm)
B
Upstream
a
b
Mitotic cell
Downstream
a/(a + b)
1.00
0.75
0.50
0.25
0.00
(1/1)
(1/1)
(1/1)
(4/4)
(1/1)
(3/4)
(2/3)
(2/3)
(1/2)
(3/6)
(1/2)
(2/5)
(1/3)
(2/8)
(1/4)
(1/4)
(0/2)
(0/1)
(0/1)
$0.00 \leq \Delta C < 0.429 \times 10^{-3}$
$0.429 \times 10^{-3} \leq \Delta C < 0.866 \times 10^{-3}$
$0.866 \times 10^{-3} \leq \Delta C < 2.497 \times 10^{-3}$
$2.497 \times 10^{-3} \leq \Delta C < 5.009 \times 10^{-3}$
Concentration gradient ΔC (nM/μm)
