# Supplementary material for: Quantitative analysis of sensitivity to a Wnt3a gradient in determination of the pole‐to‐pole axis of mitotic cells by using a microfluidic device
Source: FEBS Open Bio. 2018 Nov 9;8(12):1920–35. doi: 10.1002/2211-5463.12525 (PMC6275273; doi:10.1002/2211-5463.12525)
Supplement: Supplementary file 13 — Table S1. Parameters of mould fabrication. [file FEB4-8-1920-s013.pdf]

TABLE S1 Parameters of mould fabrication

Mask	Resist	Rotation speed	Softbake	Exposure dose	Postbake (60°C/100°C)
microgrooves	SU8 3010	500 rpm	15 min	420 mJ/cm^2	1 min / 8 min
main channel	SU8 3050	1000 rpm	15 min	480 mJ/cm^2	1 min / 5 min
under-layer channel	SU8 3050	1300 rpm	20 min	420 mJ/cm^2	1 min / 8 min
upper-layer channel	SU8 3050	1300 rpm	20 min	480 mJ/cm^2	1 min / 10 min
air valve[a)]	SU8 3050	1300 rpm	20 min	480 mJ/cm^2	1 min / 10 min

[a)] To produce a mould with a height of 200 μm, spin coating and softbake processes were performed twice.
